# In foreign pastures: Livestock mobility at Hazor and the permeability of Iron Age geopolitical borders in the Southern Levant

**DOI:** 10.1371/journal.pone.0328934

**Published:** 2026-04-10

**Authors:** Cheryl A. Makarewicz, Sarah Martini, Nimrod Marom, Shlomit Bechar

**Affiliations:** 1 Department of Archaeological Sciences, School of Archaeology and Maritime Cultures, University of Haifa, Israel; 2 Archaeology Stable Isotope Laboratory, Institute for Prehistoric and Protohistoric Archaeology, University of Kiel, Germany; 3 Department of Anthropology, Yale University, New Haven, Connecticut, United States of America; 4 The Leon Recanati Institute for Maritime Studies, School of Archaeology and Maritime Cultures, University of Haifa, Israel; 5 Interdisciplinary Centre for Archaeology and Evolution of Human Behavior (ICArEHB), University of Algarve, Portugal; 6 Department of Archaeology, School of Archaeology and Maritime Cultures, University of Haifa, Israel; University of California Santa Cruz, UNITED STATES OF AMERICA

## Abstract

Southern Levantine societies during the Iron Age II (10^th^–8^th^ centuries BCE) witnessed the formation of competing territorial polities and urban revival following a period of settlement ruralization and dwindling regional exchange economies associated with the collapse of Late Bronze Age Canaanite city-states around 1200 BCE. Recent work has revealed diverse expressions of complex political organization, state-sponsored cultic activity, and inter-polity conflicts within the region during the Iron Age II, but the impact of regular military confrontations and ensuing territorial reconfiguration on agro-pastoralist economic systems that supported these polities is unknown. Here we explore inter-polity border dynamics between the Israelite and Aramean states during periods of conflict (Iron Age IIA) and concord (Iron Age IIB) by establishing landscape-use strategies involving the most mobile element of Iron Age subsistence and production systems – domesticated sheep and goats, at Tel Hazor located in the Hula Valley, a place of direct contact between Aram and ancient Israel. Multi-stable isotopic analyses of bovid livestock teeth indicate agro-pastoralist herders grazed their animals in well-watered pastures locally near Hazor and also further afield in the Golan Heights. The continuous use of spatially diverse pasturing regimes throughout the Iron Age II suggests household-based agro-pastoralist land use transcended regional political discord. Everyday movement of herders and their flocks to distant pastures was not restricted despite conflict between the military and ruling elites of the Israelite and Aramean states, suggesting that borderlands between states were permeable.

## Introduction

Ancient borders and borderlands shaped how states enacted hegemonic power internally and externally, engaged in military actions, participated in exchange networks, and directed their own subsistence production. Such demarcated places may have included spatially diffuse areas claimed by competing states as their own territory, neutral buffer zones between two states, zones of intersection between hinterlands linked to different, cooperating or competing urban cores, or hard boundaries demarcated by physical barriers− either human built or distinctive topographical features, that impeded cross-polity movement and facilitated the exercise of political and military power over the landscape (e.g., the Roman *limes*) [1–3]. Despite the role of borders in shaping cross-polity interaction at various scales between households, communities, and states, it remains to be seen the impact, if any, of state borderlands beyond political and military activities. Much attention has been paid to how borders, frontiers, and borderlands defined and influenced political interaction, trade relations, alterity, and the movement of people and goods [1,4–6], but less so on the subsistence activities carried out by communities and households. There is similarly scant information on how ancient borders and borderlands restricted or encouraged agro-pastoralist production that supported the state apparatus.

**Fig 1:**
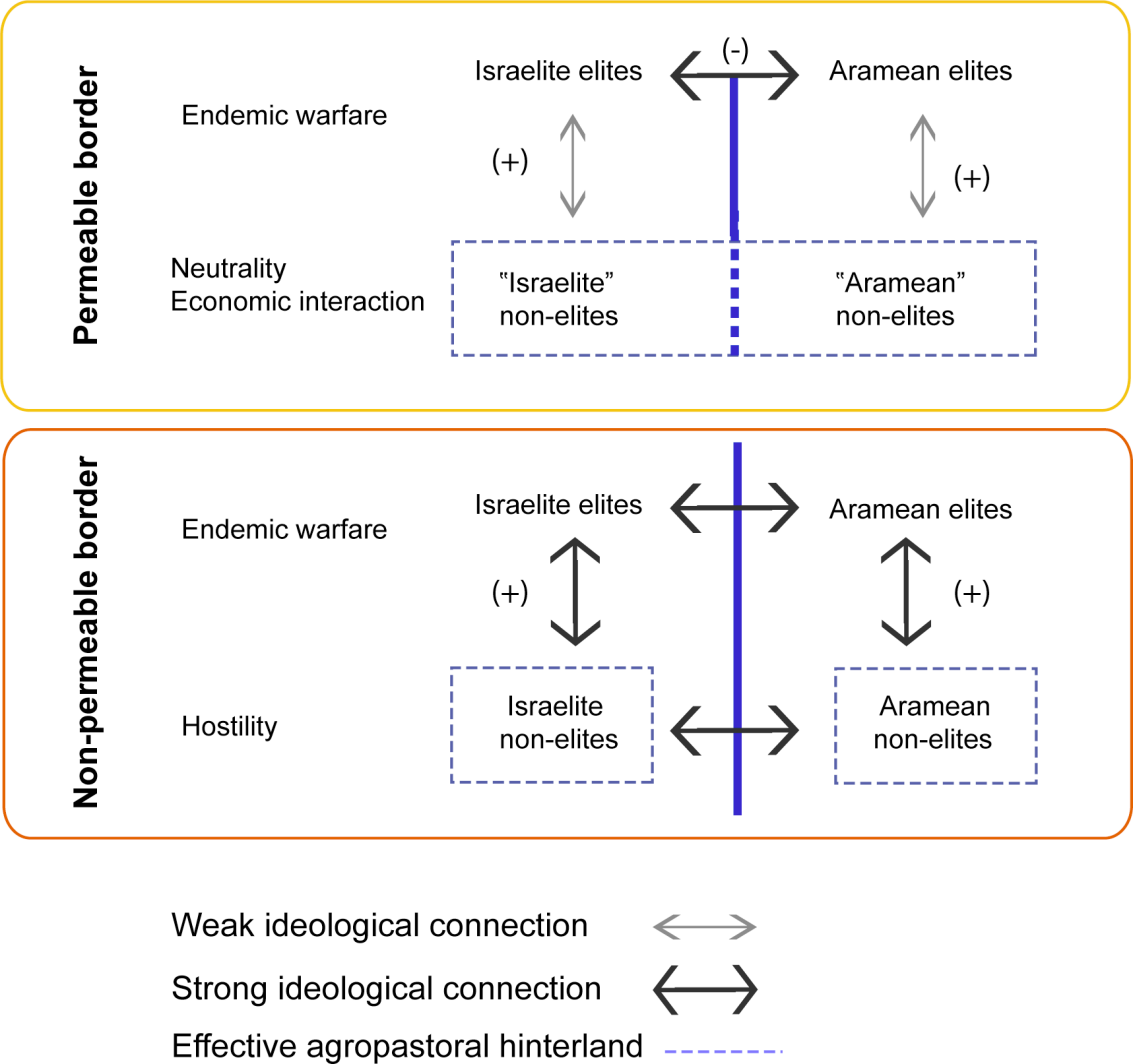
Schematic illustration of relationships between communities belonging to conflicting polities, contrasting a permeable border model (top) and non-permeable border model (bottom). (+)/(−) represent cooperative/hostile ideological connections.

In addition to revealing different modes of inter-state relations, isolating mobility dynamics potentially informs on the degree of vertical integration *within* ancient states. Curtailed movement of people, goods, and activities associated with a particular economic segment or production cascade, for example, would create different social, economic, and political outcomes than controlled movement of elites who exercised authority over a local urban political apparatus (Fig 1). One such group of non-elite individuals whose mobility patterns could be influenced by different border dynamics are herders, whose livelihood depends on moving their livestock to plentiful pastures.

Under conditions of a *permeable border*, conflict encompasses political violence between established power groups (i.e., ‘elites’), with no percolation of the concept of a ‘dangerous other’ to people distant from ruling and military circles. Livestock and agricultural goods freely moved across borderlands, stopped intermittently by active warfare every few decades. In such circumstances, access to pastoralist infrastructure including seasonal pastures and water sources was conducted independently of the state, regulated instead through inter-community negotiations and traditional tribal arrangements, cross-cutting national boundaries.

When a *non-permeable border* is in place, quotidian every-day socio-economic interactions across borderlands are strongly curtailed across all sectors of society. Furthermore, non-permeable borders may imply the existence of a vertically integrated territorial society in which conflictual ideologies filter down to the agrarian hinterland to such an extent that much of the flow of goods between hostile territories stops. Bound to ideologies legitimizing the indigenous state, as much as they might be constrained by physical conflict, agro-pastoralists no longer enter subsistence goods and capital into regional exchange networks. Accordingly, access to pastoral resources that are distributed along geographical and environmental clines is curtailed when a non-permeable border is maintained, with state-level political dynamics superseding traditional pasture access agreements. A shift from a permeable to non-permeable borderland could result, for example, in a zooarchaeologically visible shift from mobile sheep pastoralism, which requires transhumant movement between high-quality pastures and predictable access to water, to goat husbandry that does not require high-quality forage and, thus, can be more readily conducted in a circumscribed territory.

**Fig 2:**
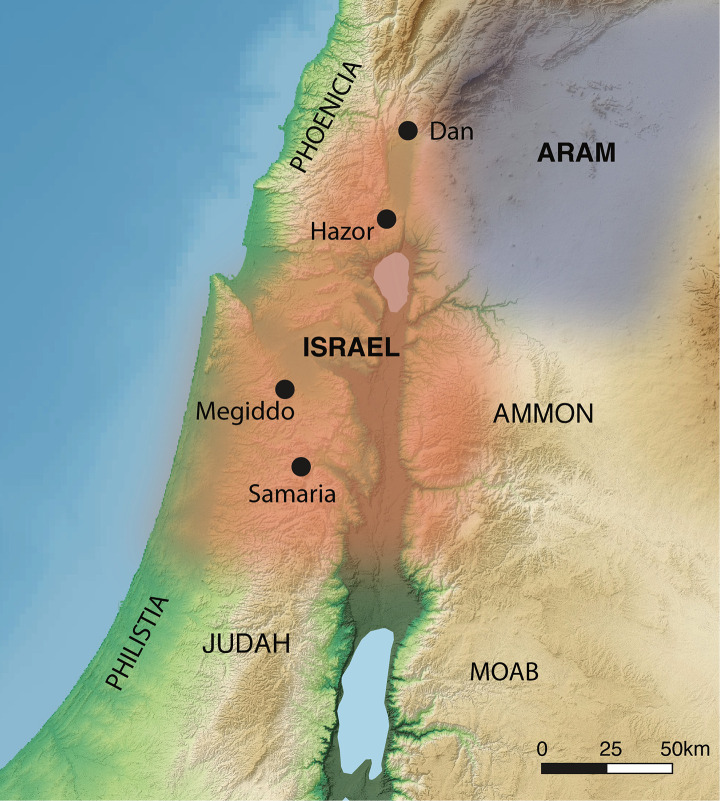
Location of Iron Age II polities in the southern Levant (following ref [11]). Estimated geographic extent of polities discussed in this text indicated by color shading: Israel = red, Aram = light gray. OpenStreetMap contributors. 2015. Planet dump [SRTM30 Colored Hillshade and Topographic WMS by Terrestris from 2022]. Retrieved from https://planet.openstreetmap.org. The lake vector was made with Natural Earth [ne_10m_lakes]. Free vector and raster map data are available at naturalearthdata.com.

The Iron Age II (IAII) of the southern Levant (10^th^–8^th^ centuries BCE) provides an opportunity to better understand the articulations between ancient border dynamics, landscape use, and livestock pasturing strategies. The Iron Age II saw the emergence and subsequent consolidation of multiple territorial polities (Fig 2), each of which exhibited an unusually strong internal cultural cohesion and ascriptive identity, several hundred years after the collapse of Late Bronze Age Canaanite city-states and palace economies that previously controlled the southern Levant [7]. These various polities, separately governed by the Phoenicians, Neo-Hittites, Philistine, Israelites, and Arameans, appear to have emplaced borders, alluded to in historical texts and thus assumed in archaeological models describing Iron Age II political organization. While biblical narratives and other textual evidence tell of endemic warfare between these Iron Age II polities in the centuries between the fall of the Egyptian empire in the 12th century BCE and the occupation by the Neo-Assyrian Empire in the second half of the 8th century BCE [8–10], there is no mention in these various records of outright borders or borderlands where political claims on a landscape may have been reduced, negotiable, or relatively unimportant.

On-going conflict between the kingdoms of Aram and Israel during the early first millennium BCE shaped regional exchange networks and subsistence production alike. We examine borderland dynamics between these two states through investigation of landscape-use strategies engaged by agro-pastoralist herders, focusing on the diversity and spatial extent of livestock pasturing regimes in contested landscapes further dissected by political borders. Focusing on domesticated sheep and goats that provided households and cities with both subsistence and raw materials that could be transformed into commodities (i.e., wool), we analyzed the strontium (^86^Sr/^87^Sr), carbon (δ^13^C), and oxygen (δ^18^O) isotopes of incrementally sampled caprine teeth in order to establish the relative extent of mobile herding activities and attendant pasturing strategies, taking advantage of predictable geospatial distribution in bioavailable strontium isotopes, oxygen isotopes in meteoric water, and also carbon isotopes at the floral base of the foodweb across the varied environments of the southern Levant. We specifically investigate if pasturing regimes in the Hula Basin (upper Galilee region) were geospatially concentrated around Israelite urban centers during the 10^th^–9^th^ centuries BCE (IRIIA) when conflict between the Israelite and Aramean states peaked and was followed by more extensive pasture access during first half of the 8^th^ century BCE (IRIIB) when conflict subsided and the hinterlands were controlled by the Israelites.

We use faunal remains recovered from Tel Hazor, located in the contested Israelite-Aramean border area in the Mediterranean landscapes of the Upper Galilee region, to empirically test two contrasting models for early inter-polity boundaries as they existed during the 10^th^–8^th^ centuries near Hazor. Under the *non-permeable border* model, conflict around Hazor in the 10^th^-9^th^ century BCE (IRIIA) would result in limited access to pastures over a relatively small catchment area suggestive of established borders that served as a bulwark against the movement of people and goods between the Israelite and Aramean states thus hindering movement of livestock across the landscape. Under a *permeable border* model, livestock mobility would be similar across the early first millennium BCE, suggesting that borders were relatively porous during Aramean and Israelite conflict and also during later Israelite political consolidation. In this case, animals and agricultural goods liberally moved across territories, suggesting that warfare between kingdoms did not have significant effect on household and communities distant from political and military centers. The alleviation of conflict around Hazor in the 8^th^ century BCE would result in expanded access to pastures over a larger catchment area, thus stimulating livestock mobility in order take advantage of more varied and higher-quality pastures.

### Tel Hazor in the Iron Age

The borderlands of Israelite Hazor changed hands between Aram and Israel throughout the 10^th^ – 8^th^ BCE centuries. The border between these two states was most probably located around the Yarmouk River, but the Hula Valley, as the area of direct contact between Aram and Israel, was likely also a contentious borderland zone [12]; the upper Jordan Valley was likely part of the Israelite kingdom [10,13]. The Israelite city of Hazor, built as a fortified urban center in the 10^th^ century BCE, functioned both as a border fortification against the Aramean state and central settlement that supported extensive domestic quarters and some administrative buildings [14,15]. Conflict erupted between both kingdoms at the end of the 10^th^ century BCE (IRIIA), after Israel separated from Judea, when the Aramaeans attacked Israel on behalf of Judea (1 Kings, 15:18–20). Although it is unclear how long the Aramaean occupation over the Israelite territories lasted, the establishment of the Omride dynasty in 881 BCE consolidated power in the Israelite state. The Omrides held the highlands of Samaria, the lower and upper Galilee, and a small portion of the Transjordan Plateau through a network of newly constructed palatial architecture placed in various cities; these buildings served to bring together diverse groups while simultaneously broadcasting their own political authority [16].

The new political authority in ancient Israel established in 842 BCE quickly succumbed to Assyrian attackers in 841/840 BCE who also engaged the Aramaean kingdom. The cessation of military activity by the Assyrians around 836 BCE, who then focused their efforts on western campaigns, opened an opportunity for the re-opening of direct conflict by the Aramaeans against the Israelites. The Tel Dan stele, for example, indicates the Aramaean king Hazael conquered the entire northern Jordan Valley [10,13]. At some point during the 9^th^ century BCE, Hazor was transformed into a large administrative center, having doubled in size and domestic buildings replaced by public buildings, which included at least five large storage buildings and two granaries thought to have supplied both the city and residents beyond. Substantial investment in a subterranean water system also took place at this time, providing the city with the means to withstand a long-term siege [14,15,17]. The rapid growth of Hazor might indicate a demographic bulwark established by the Israelite kingdom along the Aramean border in response to heightened political unrest between the two entities or, alternatively, reflect a general increase in the population suggested by shifts in regional settlement patterns (see below, section 2.2.2). The subsequent devastation of the Aramean state by the Assyrians around 803/796 BCE led to a calm period for the Israelite state, as the Arameans were no longer able to exercise autonomous political power in the region [8].

Corresponding with the decline in conflict with the Aramean state, the built environment of Hazor underwent significant changes in the 8^th^ century BCE (IRIIB) when several of the public buildings, including all the granaries and storage buildings, went out of use and were replaced by new domestic buildings and workshops. Storage facilities were identified in some domestic buildings, indicating storage was no longer conducted in public buildings but instead performed within households. Such a change in storage mechanisms at Hazor may indicate a broader change in subsistence modes, probably agricultural activities, engaged by the local inhabitants of the city. Alternatively, this may reflect a shift in the acquisition and (re)distribution of food commodities that coincided with a change in local political structures, a process suggested by the dissolution of administrative buildings and their replacement by domestic buildings and workshops. These changes in architectural construction and use within Hazor were accompanied by considerable settlement shifts in its hinterland regions. The Upper Galilee, the northern Golan Heights and the northern part of the Hula Valley experienced a sharp decrease in the number of settlements during the transition from the 9^th^ century (IRIIA) to the 8^th^ century (IRIIB) BCE [18,19]. Settlement abandonment was particularly pronounced in the mountainous regions of the Galilee, which may have served as a buffer zone between the kingdom of Israel and the Phoenician coast [18]. In contrast, the southern part of the Hula Valley and the central and southern Golan Heights experienced a dramatic increase in settlement density, perhaps reflecting the growth of Hazor [19,20], now wealthier and stronger. More settlements sprung up around the city, hoping to take advantage of economic opportunities that created trade and wealth. It may also be that northern communities relocated from potentially volatile and contested terrains to the more politically stable area surrounding Hazor, which was still under Israelite control, and, by relocating to this area, they were able to seek protection from Hazor.

The shifting political relations between Aram and Israel certainly affected trade and economic relations between and within each kingdom, but the extent to which political conflict, engaged largely by the ruling elite and military circles, was projected onto the hinterland landscapes remains largely unknown. How the economic practices of domestic households, including agro-pastoralist herders who husbanded livestock for their own subsistence and profit, were affected is not documented, nor are shifts in extensive pasturing systems strategies most affected by borderland political dynamics. Hazor’s positioning in the contested landscapes proximal to the Hula Valley makes the settlement ideally suited to examine the permeability dynamics of the Israelite−Aramean border throughout the Iron Age, when political establishments were repeatedly reconfigured by indigenous and foreign influences.

## Materials and methods

### Isotopic foundations for establishing ancient pasturing regimes

Where herders direct their livestock for pasturing depends on complex intersection of factors, including seasonally driven changes in pasture graze quality and abundance, location of pastoralist infrastructure such as water and shelter [21], pasture access rights [22], and directives from institutionalized authorities [23]. Multi-stable isotopic analyses of ancient animal skeletal tissues provide a robust means to trace livestock dietary change and mobility at high temporal and geospatial resolutions, information that further supplies new insights into the spatial dynamics of pastoral landscape use [24].

### Carbon (δ^13^C) isotopes

The carbon isotope ratios of herbivore tooth bioapatite reflect those of ingested plants, providing a means to directly document seasonality in the carbon isotopic composition of local vegetation communities and thus enabling reconstruction of fodder provisioning and livestock pasturing practices associated with mobile herding regimes [25,26]. The carbon isotope ratios of plants are determined by photosynthetic pathway, aridity, and water availability [27]. C_3_ and C_4_ plants globally average −26‰ and −12‰ in δ^13^C, respectively [28]. C_3_ plants are sensitive to water stress, closing their stomata under conditions of reduced water availability. This physiological response decreases photosynthesis, leading to a decrease in carbon fixation from CO_2_, which in turn enriches leaf tissue growth approximately 2 − 5‰ in ^13^C [29,30]. The carbon isotopic composition of semi-arid environment floral growth shifts seasonally with warmer season growth enriched in ^13^C relative to cooler wet seasonal growth [31,32]. C_3_ plants are also sensitive to arboreal cover density. The combination of decomposing leaf litter, recycling of atmospheric CO_2_, and/or low light levels that slow photosynthesis rates creates a ‘canopy effect’ that can lower understory floral δ^13^C values up to 5‰ [33,34]. We apply a enamel bioapatite–diet isotope enrichment factor of 14.1 ± 0.5‰ to estimate dietary δ^13^C values for bovid livestock [35].

### Oxygen (δ^18^O) isotopes

The geospatial distribution of environmental oxygen isotopes is broadly predictable across landscapes, thereby providing a means to examine animal mobility. The oxygen isotopic composition of precipitation is influenced by rainfall amount, temperature, and humidity [36,37]. In semi-arid environments, seasonal variation in temperature, precipitation levels, and aridity produces corresponding seasonal shifts in precipitation oxygen isotope values, with warmer season meteoric waters typically enriched in ^18^O due to higher temperatures, increased aridity, and reduced rainfall [36]. Precipitation δ^18^O values are also influenced by altitude with ^18^O-depleted meteoric waters falling on cooler, high elevation locations less impacted by evaporative fractionation [36]. Meteoric water δ^18^O values decrease approximately 0.3‰ per 100m increase in elevation [38,39].

Leaf water oxygen isotopes reflect in part the oxygen isotopic composition of meteoric waters [40,41], but are strongly influenced by soil water δ^18^O values, rooting depth, leaf structure, and evapotranspiration rates [42]. Evaporation at the ground-air interface results in ^18^O enrichment of upper layer soil waters relative to meteoric water [43]. Fractionation effects are minimal during transport of soil water via non-transpiring plant parts such as roots and stems [31,44]. Substantial ^18^O enrichment of plant water does occur, however, at evaporation sites in leaves [45,46], from which the ^18^O-enriched water diffuses throughout the leaf [42].

The δ^18^O values of herbivore tooth enamel carbonates are influenced by the oxygen isotopic composition of imbibed water which includes open water sources, groundwater, and leaf water [47,48]. Obligate drinkers, animals such as cattle that require substantial quantities of water on a daily basis, exhibit lower δ^18^O values than non-obligate or semi-obligate drinkers that meet their water needs from ^18^O-enriched leaf water [49]. Furthermore, herbivores ingesting a higher proportion of grass exhibit higher δ^18^O values compared to animals consuming higher amounts of browse [25]. Shallow-rooting grasses exhibit pronounced evaporative effects across the leaf surface [50] so that ingestion of ^18^O-enriched leaf water by sheep and goats would result in higher δ^18^O values compared to obligate drinking cattle.

### Strontium isotopes

Strontium isotopes in biogenic tissues including enamel hydroxyapatite provide a robust means to evaluate animal mobility at local, regional, and transregional scales [51–53]. The distribution of bioavailable strontium in soils varies across a defined region, determined by the composition of local weathered bedrock as well as strontium delivered by aeolian dusts and rainfall [54,55]. Strontium isotope ratios (^87^Sr/^86^Sr) of different bedrock geologies are defined by substrate age and the original ratio of rubidium and strontium present in bedrock [54]. Strontium isotopes enter the foodweb primarily through plants which take up resident strontium present in soils without isotopic fractionation due to the heavy molecular weight of strontium isotopes [56]. Herbivores thus directly incorporate strontium isotopes derived from graze and, to a lesser extent, water [57]. Unlike bone, which in general does not retain *in vivo* strontium isotope ratios [58], the highly organized crystalline structure of tooth enamel is highly resistant to diagenetic alteration, providing authentic values associated with the period of enamel mineralization [59].

### Southern Levantine environments in the Upper Jordan Valley

Hazor is situated in the Hula Basin (70 m a.s.l.), which constitutes the immediate hinterland and periphery of Hazor (Fig 2). Part of the Upper Jordan Valley (UJV), the Hula Valley covers 180km^2^. To the west, the UJV is bounded by the Upper Galilee Mountains (1000 + m a.s.l.) composed of Cretaceous limestone and Neogene sedimentary rocks. To the east of the UJV lies the Golan Heights, reaching over 1000 m a.s.l and made up of successive layers of Pliocene and Pleistocene volcanic bedrock [60]. Basalts and pyroclast comprise the geology to the south/southeast of Hazor (north of Lake Kinneret). The distribution of bioavailable strontium in the soils of the Upper Jordan Valley, Golan Heights, and Galilee is determined by the composition of locally weathered bedrock and strontium from aeolian dusts, largely originating from the Sahara, as well as by precipitation moving bioavailable exchangeable strontium from marine deposits and sea spray [61,62].

**Fig 3:**
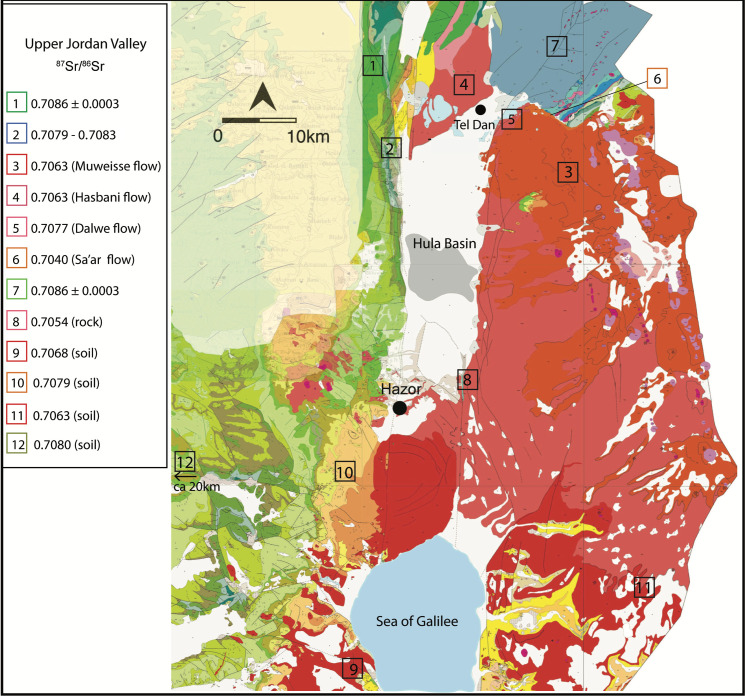
Geological map of the Upper Jordan Valley and Lower Galilee [60]. Jurassic limestones **=** blue, Cretaceous dolomites and limestones **=** green, Eocene limestones **=** orange, Neogene conglomerates **=** yellow, Pliocene and Pleistocene cover basalts **=** red. Soil and rock ^87^Sr/^86^Sr ratios from ref [120]. Estimated ^87^Sr/^86^Sr ratios for Jurassic limestones associated with terra rossa soils (green box 1) (displayed in ref [109]). Bioavailable plant ^87^Sr/^86^Sr isotope values are indicated for terra rossa soils (green box 1), rendzinas (blue box 2), and basalts (red **box 3 – 6**) (original data from ref [63] and also displayed in ref [109]). Dissolved strontium in Banias spring water derived from the Jurassic unit yields a ^87^Sr/^86^Sr ratio of 0.7072 [64].

Differences in bedrock geology, associated soil members, deposition of atmospheric strontium, and mean annual precipitation together produce spatial distinction in bioavailable strontium isotopes (^87^Sr/^86^Sr) in the hinterlands surrounding Hazor [63]. The Upper Galilee Mountains are dominated by terra rossa soils derived from the weathering of underlying Cretaceous limestones and dolomites, yielding ^87^Sr/^86^Sr ratios of 0.70831 to 0.70883. Rendzina soils, comprised of calcareous soils developed from Cretaceous and Neogene chalks and marl, yield lower strontium isotopes ranging from approximately 0.70790 to 0.70840 [63]; rendzinas occur west and southwest of Hazor, developed over Eocene limestone bedrock (Fig 3). Golan basalts exhibit low strontium isotope values ranging from approximately 0.70448 to 0.70770, with the youngest flows dating to the late Pleistocene yielding the lowest values, and flows dating to the upper Pliocene exhibiting the highest values [63].

The Hula Valley is situated within the Lake Kinneret watershed (2570 km^2^), which includes the lower-elevation topographic drainage of the upper Jordan River that flows directly into Lake Kinneret, as well as an upper watershed defined by the karstic limestone mountain ridges of Mt. Hermon. There is a strong precipitation cline across the southern Levant along an east-west and north-south gradient. Strong orographic effects contribute to the precipitation gradient over the Lake Kinneret watershed, where rainfall amounts range from 400 mm/year for Lake Kinneret to 1200 mm/year for Mount Hermon [65,66]. Precipitation falls during the cooler seasons (November to April) with higher precipitation levels at higher elevations. Mean annual precipitation in the Hula Valley is 500–600 mm/year. Summers are hot and dry with annual temperatures averaging 27ºC in the Hula Valley and 21ºC for the Upper Galilee.

Hazor is situated within a seasonal C_3_ Mediterranean phytogeographic zone, relatively close to other phytogeographic zones characterized by different vegetation communities. C_4_ sedges growing around springs and ponds are also present in the Mediterranean environments of the UJV [67]. Higher precipitation levels in the Golan support C_3_ growth depleted in ^13^C particularly during the wet season [32]. A drier Mediterranean facies that also supports some Irano-Turanian flora spreads south of Lake Kinneret and along the mid-elevation landscapes of the Rift Valley, while a more arid Saharo-Arabian zone, also containing some Irano-Turanian vegetation including ^13^C-enriched C_4_ taxa, is distributed along lower elevations [68]. The very lowest elevations of the Rift Valley averaging −200 to −300 m b.s.l. receive less than 200 mm of rainfall per year, supporting Saharo-Sudanian and Sudan-Zambezian phytogeographic zones that contain a higher abundance of C_4_ flora [69].

### Zooarchaeological methods

Zooarchaeological analyses of faunal remains from Hazor provide a means to evaluate herd management strategies that may articulate with particular pasturing practices and Aramean-Israelite political dynamics. The faunal remains from Area M were recovered from accumulations of urban refuse representing time-averaged secondary discard associated with storage buildings, domestic structures, and also general fill contexts (Table 1). Not unexpectedly, no animal pens have so far been identified inside the built environment. Livestock may have belonged to Hazor households, kept outside the city and managed through kin-based or hired labor, or owned by pastoralists extrinsic to the urban community at Hazor. Regardless if livestock herds were husbanded by urban or rural components of Israelite society, successful caprine husbandry requires regular access by herds to sizeable pastures, and caprines consumed in the city would reflect the isotopic inputs related to pasturing regimes enacted on herds.

**Table 1:** Contextual information for mandibular tooth specimens sampled for isotope analyses. O = *Ovis aries*, C = *Capra hircus*, OC = Ovis/Capra. ‘n’ indicates number of increments measured for each tooth. Mandibular wear stage determined following ref [76] and age at death estimated using ref [86]. Age cohorts: Age 1 (MWS A-C, 0–12 months; Age 2 = MWS D, 1–2 years; Age 3 = TWS E, 2–3 years; Age 4 = MWS F, 3–4 years; Age 5 = TWS G-I, 4–10 years).

ASIL #	Hazor #	Taxon	Context	Context Description (BCE)	n	Mandibular Wear Stage	Estimated Age Cohort at Death
**IRIIA**							
5816	76883.2	O	L-11-362	10th century pit	18	e-g	4
5750	78976	Bos	L15-310	9th century fill	13	e-j	4
5751	B79071	OC	L15-325	9th century fill	23	b-d	3
5752	79017.1	OC	L15-325	9th century fill	23	b-d	3
5753	75605.1	C	L10-327	9th century installation	13	e-j	4
5756	75520	C	L10-320	9th century installation	7	e-g	4
5757	70807	C?	L15-311	9th century fill	20	b-d	3
5805	75697	O	L10-310	9th century floor of storage building	18	e-g	4
5808	75757	O	L10-331	9th century floor of storage building	14	e-g	4
**IRIIB**							
5754	73170	Bos	L08-316	8th century fill	10		4
5758	73199	OC	L08-316	8th century fill	12	e-j	4
5801	73761.1	OC	L08-316	8th century fill	13	a	2
5812	73485	OC	L08-316	8th century fill	10	e-g	4
5813	73288	OC	L08-316	8th century fill	15	e-g	4
5817	73363.2	Gazella	L08-316	8th century fill			
5820	73698	OC	L08-316	8th century fill	15	f	4

Faunal remains were collected by hand during the excavation and were identified using the comparative collection of the Laboratory of Archaeozoology at the University of Haifa. Recovery by hand was complemented by extensive wet-sieve sampling, which yielded very few mammal bones that were taxonomically or otherwise informative, suggesting that hand recovery provides as good a sample of the faunal record for medium- and large-sized mammals (cf. ref [70]). Faunal remains are archived in the Israel Antiquities Authority while teeth analyzed for carbon and oxygen stable isotopes as part of this study are archived in the Archaeology Stable Isotope Laboratory (ASIL) at the University of Kiel. To estimate taxonomic and demographic variables of livestock use, we used a focused protocol that records a set of data-rich diagnostic zones [71], (see discussion in [72–74]). Epiphyseal fusion [75], tooth eruption and wear [76], and biometrical [77,78] data were collected when possible for all livestock taxa (S1 Table and S2 Table). To check the equivalence of taphonomic deposition scenarios between contexts, data on bone surface modification such as weathering, carnivore gnawing, butchery, and burning marks were noted when observed with the naked eye.

Statistical analyses were carried out in R (R Core Team 2018) and visualized using the ggplot2 package. The association plot was drawn using the “vcd” package v. 1.4–7 [79]. Least-square regression of cosine functions was conducted in Excel using a file published by Pieterse [80].

### Selection of teeth and intra-tooth sampling for isotope analysis

Teeth do not remodel after mineralization, preserving a seasonal-scale record of dietary intake and environmental inputs. We selected third mandibular molars for our analyses because these specimens were the most well represented in the assemblage. For sheep and goats, formation of the third mandibular molar begins at approximately 9–10 months and is completed by 20–22 months [81,82]. Crown formation and enamel mineralization rates in goats and sheep appear to be broadly similar [83]. The third mandibular molar exhibits greater temporal variation in crown formation rates between individuals compared to the second mandibular molar, although the amplitude of seasonal isotopic change captured in the tooth crown is similar [84]. For cattle, the third mandibular molar begins formation at approximately 9–10 months in age and is completed by ca. 23–24 months [85]. Continuous wear of hypsodont bovid teeth erases some portion of seasonal isotopic inputs recorded in tooth enamel. Consequently, the amplitude of intra-tooth isotopic change visible in older animals is reduced due to elimination of seasonal peaks and troughs within the sequence (i.e., minimum and maximum isotope values). Mandibular wear stages used to assess relative animal age at death, described above, were used to estimate the extent of tooth structure loss of sampled specimens (Table 1).

All necessary permits were obtained for the described study, which complied with all relevant regulations. Archaeological tooth samples were exported to the Archaeology Stable Isotope Laboratory (ASIL) at the University of Kiel under permit number 14197 issued by the Israel Antiquities Authority. Third mandibular molars (M/3) from domesticated sheep (*Ovis aries, n = 2*), goats (*Capra hircus, n* = 3), sheep/goat (Ovis/Capra, *n* = 2), and cattle (*Bos taurus,* n =1) were selected from early Iron Age II (IRIIA) contexts dated to approximately the 10^th^–9^th^ centuries BCE, a period of conflict between the Aramean and Israelite states. This chronological assignment is based on the stratigraphic context of the finds and ceramic typology of pottery sherds recovered from the same stratigraphic context as the faunal remains. M/3’s from five sheep/goat (n = 5, *Ovis*/*Capra*) one gazelle (n = 1, *Gazella* sp.), and one cattle (n = 1, *B. taurus*) were selected from the late Iron Age II (IRIIB) contexts dated on the basis of stratigraphy and ceramic topology to the 8^th^ century BCE, a period defined by a cessation of conflict between the two polities reflecting the devastation of the Aramean state following the Assyrian conquests. Third mandibular molars were identified as belonging to either sheep or goat based on morphological criteria established by ref [87].

Teeth were cleaned mechanically using a scalpel in order to remove both dental calculus and any adhering sediments. Teeth were then sonicated in distilled water to ensure full removal of contaminating particles and dried overnight at 60^o^C. A sequence of horizontal bands approximately 1 mm in width were drilled perpendicular to the growth axis from the tooth crown (earliest forming) to the enamel root junction (ERJ; latest forming) on the buccal side of the anterior tooth loph using a diamond-tipped drill bit. Sampling the same tooth loph enhances comparability of isotopic time-series between individuals by reducing the impact of tooth geometry on the shape and amplitude of intra-tooth isotopic change [88–90]. Overall, the seasonality of intra-tooth isotopic change recorded in hypsodont teeth is attenuated relative to seasonal change in environmental isotopes due to incremental deposition of enamel matrix at daily scales, successive cross mineralization during subsequent enamel maturation over longer timescales, tooth geometry, and a sampling strategy that cross-cuts successive mineralization fronts. Amelogenesis progresses through multiple, discontinuous stages during which a primary matrix is laid down in successive cone-like structures, subsequently mineralizes, and matures over an extended period of time [91–94]. The extended process of enamel maturation involves the deposition of secondary mineralization fronts that run transverse to the long axis of enamel prisms, introducing time-averaging in the measured isotopic sequences [95–97]. An exponential decrease in tooth growth rates towards the crown-enamel junction further integrates isotopic inputs [97–99].

Enamel powders were soaked in approximately 1 mL of 0.1 M acetic acid for 4 hours to remove diagenetic carbonates [100,101], then rinsed with distilled water and centrifuged at 6000 rpm five times. Enamel powders were then freeze-dried. After treatment, samples were reacted with 100% orthophosphoric acid at 75ºC in an automated cryogenic distillation system (Kiel IV device) interfaced with a ThermoScientific MAT 253 mass spectrometer located in the Leibniz Laboratory for Radiometric Dating and Stable Isotope Research at the University of Kiel. Two international carbonate standards, NBS-19 and IAEA-603, were run daily. Analytical precision was 0.05‰ for carbon isotope ratios and 0.08‰ for oxygen isotope ratios. Three different internal enamel standards measured an analytical precision of 0.10‰ for oxygen isotope ratios and 0.15‰ for carbon isotope ratios (CM1_D; 48 measurements), 0.08‰ for oxygen isotope ratios and 0.14‰ for carbon isotope ratios (ER1_C; 8 measurements), and 0.07‰ for oxygen isotope ratios and 0.09‰ for carbon isotope ratios (ER1_D; 40 measurements). All isotopic values were reported relative to the V-PDB (Vienna Pee Dee Belemnite) standard using NBS-19.

Enamel samples for strontium isotope analyses were removed from portions of the tooth exhibiting high summer season maximum δ^18^O values and low winter season minimum δ^18^O values. For strontium isotope analysis, separation chemistry for enamel samples was performed in a class 100 clean lab facility located in the MC-ICP-MS facility in the Department of Geological Sciences, University of Cape Town (UCT). Samples were transferred to Savillex beakers digested in 2–3 mL of 65% HNO_3_ at 140ºC. After complete dissolution, the samples were dried overnight at 130ºC and re-dissolved in 2 mL 2M HNO_3_ and loaded into 800 μL Teflon columns containing pre-conditioned Eichrom Sr-spec pure resin (500 = 100 μm). After six washes with 2M HNO3, the Sr was eluted with 3 mL of ultrapure deionized water, dried, and re-dissolved to a volume of 0.2% HNO_3_. ^87^Sr/^86^Sr isotope analyses were performed using a Nu Instruments NuPlasma HR multi-collector ICP-MS instrument housed in the Department of Geological Sciences, University of Cape Town. ^87^Sr/^86^Sr data presented were referenced to bracketing analyses of the international strontium isotope standard NIST SRM987 using a ^87^Sr/^86^Sr normalizing value of 0.710255. Isobaric interference of ^87^Rb on ^87^Sr was corrected using the measured signal for ^85^Rb and the natural ^85^Rb/^87^Rb ratio. The effect of instrumental mass fractionation was corrected using the exponential law and a ^86^Sr/^88^Sr value of 0.1194. All samples were run to an internal precision of ± 0.000037 (2SE) or better. Repeat analyses of an in-house carbonate reference material (NM95) analyzed as an unknown along with the enamel samples during processing of samples for this study yielded an ^87^Sr/^86^Sr value of 0.708910 ± 0.000030 (n = 3) in agreement with long-term data for this material (0.708911 ± 0.000040 2s; n = 414; over >8 years). Total procedural strontium blanks were typically better than ~250 pg and therefore negligible.

### Modelling birth seasonality

The position of maximum δ^18^O and δ^13^C values in the tooth crown relative to the ERJ provides information on the seasonality of birth and dietary intake, respectively. However, inter-individual variability in tooth size and geometry impacts the shape of isotopic curves and, in turn, influences the relative positioning of minimum and maximum isotope values in time series [102]. Here, we reduced the impact of inter-individual variation in tooth size to better fit isotopic time-series alignments by normalizing distances of sampled increments using an equation derived from a cosine function developed by ref [103]. Although the M/3 exhibits wider variation in growth rate than the M/2 [102,103], third mandibular molar-based calculations will also separate season of birth between individual animals [84,104]. Previous work comparing oxygen isotope curves modelled for M/2 and M/3 teeth from the same analytical cohort indicates temporal disparities in modelled birth seasonality, with the second molar demonstrating a shorter birthing period of only four months while the third molars demonstrate an extended birth season taking place over six months [84].

A least square regression was used to obtain best-fit values for parameters *A*, *x*_*0*_, *X*, and *M* while the values of *p*, *b*, and *x*_*B*_ were fixed to 0 and *x*_*A*_ was fixed to 10^6^. This four-parameter model was found to be sufficient by ref [103] for modelling the majority of cases. The season of birth was determined by evaluating the position in the tooth crown where the highest (*x*_*max*_ *= x*_*0*_) and lowest (*x*_*min*_ *= x*_*0*_ *+ X/2)* δ^18^O values were modelled relative to the period of the annual cycle (*X*). The birth seasonality of individuals 5756 and 5808 from the IRIIA and 5758, 5812, and 5817 from the IRIIB could not be determined due to shortness of isotopic sequence.

## Results

### Retention of caprine management systems over time at Hazor

**Fig 4:**
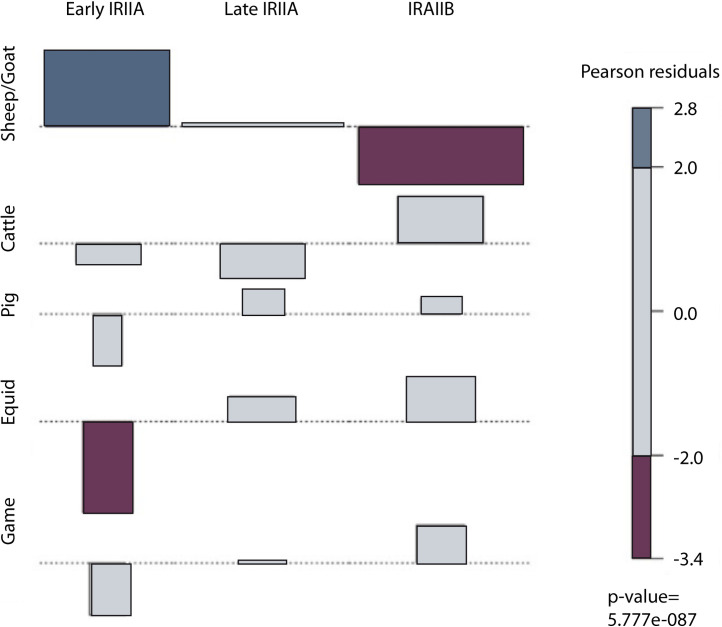
Chi-square standardized residuals of taxa by period. Dark purple = significant under representation, dark blue = significant overrepresentation. Total chi-square = 43.97, P < 0.001.

**Fig 5:**
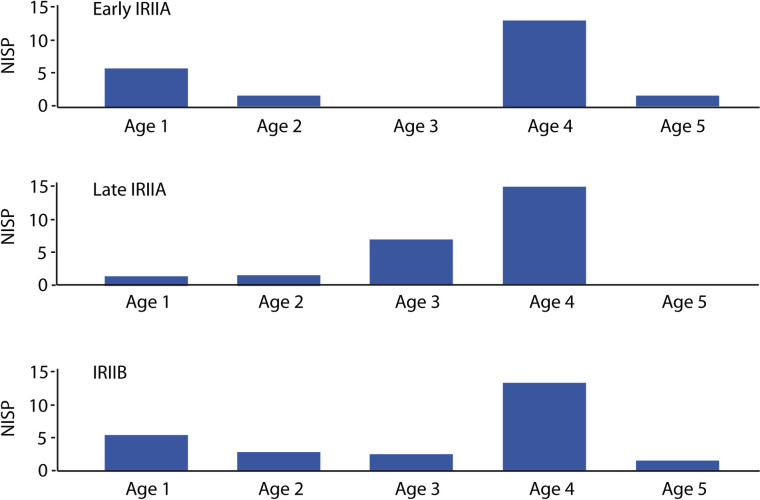
Age-at-death distributions for caprines for Iron Age II Hazor analyzed as part of this study based on ref [86] (computed according to mandibular wear stages defined by ref [76]). Age cohorts: Age 1 (MWS A-C, 0–12 months; Age 2 = MWS D, 1–2 years; Age 3 = TWS E, 2–3 years; Age 4 = MWS F, 3–4 years; Age 5 = TWS G-I, 4–10 years).

Shifts in geopolitical boundaries and border permeability, as well as subsistence and economic dynamics influence the spatial extent of pastoral systems and shape of herd management strategies that entail adjustment of livestock mobility and herd demography. Zooarchaeological results indicate similarity in faunal assemblages representing IRIIA and IRIIB occupation at Hazor. A total number of 737 specimens were identified to genus in the Iron Age II assemblage (Table 2). Sheep (*Ovis aries*) and goats (*Capra hircus*), evenly represented with a ratio close to 1:1 (Table 2, Fig 4; identifications based on ref. [105]), were heavily exploited in both periods, followed by cattle (*Bos taurus*) and equids. The latter included both donkey (*Equus asinus*) and horse (*E. caballus*), clearly distinguishable by size (Late IRIIA, 9^th^ century: 13 horse, 14 donkey; IRIIB, 8^th^ century: 13 horse, 16 donkey, see Table 2). Deer (*Dama mesopotamica*) and gazelle (*Gazella* cf. *gazella*) were occasionally hunted. Tooth wear data from sheep/goat specimens indicate focused harvesting of mature adults approximately 4 years in age. Some slaughter of yearlings was also undertaken during the early IRIIA (10^th^ century BCE; Table 1, Fig 5). The predominance of skeletal remains from mature adults is typical of a livestock producer economy and contrasts with an abundance of older juveniles and young adults expected for consumer settlements (e.g., ref [106]). Overall, strong similarity in sheep-to-goat ratio and age-at-death calculated for IRIIA and IRIIB occupation suggests no major change in caprine production goals for subsistence or otherwise between the 10^th^ and the 8^th^ centuries BCE.

**Table 2:** Relative frequencies of taxa from Iron Age II Hazor (Area M). Number of Identified Specimens (NISP) values are based on diagnostic zone counts according to ref. [71]. Early IRIIA (10^th^ century BCE), Late IRIIA (9^th^ century BCE), IRIIB (8^th^ century BCE).

Taxon	Early IRIIA		Late IRIIA		IRIIB	
	NISP	%	NISP	%	NISP	%
*Ovis/Capra*	89	81	99	63	93	53
*Capra hircus*	21		35		31	
*Ovis aries*	21		39		35	
*Bos* cf. *taurus*	24	15	38	14	64	21
*Sus scrofa*	1	1	12	4	12	4
*Equus* sp.	3	1	34	12	41	14
*Camelus* sp.	0	0	0	0	1	<1
*Canis* sp.	0	0	3	1	1	<1
*Dama mesopotamica*	3	1	10	4	16	5
*Gazella* cf. *gazella*	0	0	5	2	6	2
Total	162		275		300	

However, a decline in the frequency of caprines from 81% to 53% from the early IRIIA to the IRIIB, accompanied by a concomitant, dramatic increase in the frequency of cattle and equids, rising from 16% in the IRIIA to 35% in IRIIB, suggests an intensification in agriculture, an activity which involved a larger number of traction animals, over large-scale pastoral production (see Table 1 and Fig 4). Notably, this corresponds with a decrease in tree cover between 9^th^–8^th^ centuries BCE associated with land clearance, possibly for increased agricultural output [107]. A shift in economic strategies linked to food production is also reflected in the architectural changes of the city with large, public storage buildings falling out of use during the IRIIB, replaced by silos placed in households. Despite changes in the type of animal and agricultural production, similarity in caprine management systems with the exploitation of goats and sheep more or less similar across the Iron Age II suggests that caprine mobility patterns were not dictated by household subsistence or economic goals but by landscape accessibility, as defined by polities and borders.

### Caprine birth seasonality

**Fig 6:**
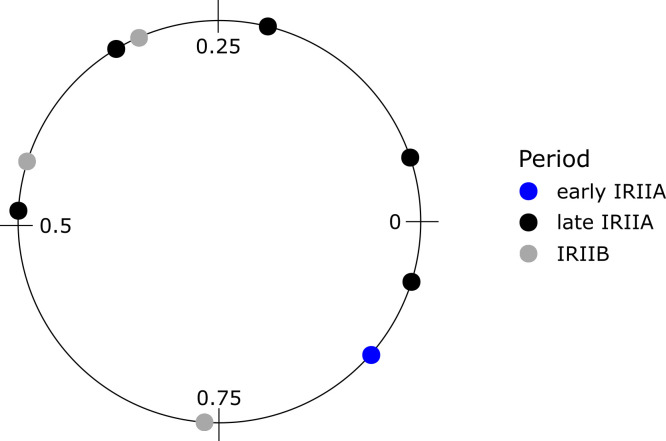
Relative positions of maxima δ^18^O values measured in intra-tooth oxygen isotope sequences of caprine third molars, normalized to the period of the yearly cycle [period (X_0_/X)], as determined using the modelling procedure according to ref [103].

Establishing the timing and distribution of animal season of birth is an important husbandry strategy that helps herders coordinate the production of milk and meat throughout the year. Birth seasonality in sheep and goats can be established through analysis of the positioning of summer-season maximum and winter-season minimum oxygen isotope values expressed in mandibular molars [102]. The locations of minima and maxima δ^18^O values relative to the enamel-root junction (ERJ) are presented in Table 3 and, based on those data, the modelled season of birth is presented in Fig 6 for Late IRIIA and IRIIB animals. For each case, the fit of the model was tested using an ANOVA; all p-values were below 0.05. Model results indicate moderate variation in caprine birth seasonality. An offset of ¼ of a full period (*X*) is visible between late IRIIA (n = 5) and IRIIB (n = 3) *x*_*max*_ and *x*_*min*_ values. When normalized to the period of the cycle, the position of maximum δ^18^O values indicates extended birth seasonality at Tel Hazor, stretching over ca 5–6 months (IRIIA: 0.54 year; IRIIB: 0.42 year; Fig 5), and that this period shifted by approximately 3 months (0.24 year) between the earlier and later Iron II occupations.

**Table 3:** Best fit (least-squares regression) and cosine function for A (amplitude), x_0_ (position of δ^18^O_max_ value in tooth crown), X (period of cycle), M (mean), x_min_ = position of lowest δ^18^O value in tooth crown; p-value refers to ANOVA test on the least-square regression. O = *Ovis aries*, C = *Capra hircus*, OC = Ovis/Capra. Absent specimens are those that did not yield clear seasonal minimum or maximum oxygen isotope values required for calculations.

Specimen	Species	A	x_0_(mm)	X(mm)	M(‰)	x_min_(mm)	x_0_/X(%)	x_min_/X(%)	p-value
**Early IRIIA**									
5816	O	2.9	26.1	29.4	2.7	11.44	0.89	0.39	2.14E-12
**Late IRIIA**									
5751	OC	2.3	18.5	37.6	−3.0	37.2	0.49	0.99	9.41E-14
5752	OC	1.8	2.0	39.3	−3.4	21.7	0.05	0.55	7.55E-09
5753	C	1.4	8.2	24.4	−0.6	20.4	0.34	0.84	0.000337
5757	C?	1.7	32.7	34.3	−0.5	15.5	0.95	0.45	8.73E-07
5805	O	2.5	8.01	38.0	−1.6	27.0	0.21	0.71	1.6E-10
**IRIIB**									
5801	OC	1.6	16.8	23.0	0.8	5.4	0.74	0.24	0.000592
5813	OC	1.0	5.9	18.8	−0.8	15.3	0.32	0.82	0.000599
5820	OC	3.6	14.1	31.2	−1.8	29.7	0.45	0.95	2.75E-09

Asynchronicity in modelled sequences likely reflects a combination of loosely staggered birth seasonality and also inter-tooth differences in third mandibular molar tooth formation rates which are more variable than the second mandibular molar more commonly used to establish birth seasonality [84]. Short lambing periods are associated with transhumant herd movement for the production of wool and meat, while extended birth seasons are associated with closely pastured animals for meat and milk. The extended birth season seen at Hazor fits well with production strategies oriented toward milk production for household consumption along with staggered meat production for markets. Extended birth seasonality observed in both the IRIIA IRIIB period could also reflect *ad hoc* provisioning of Hazor with live animals or meat obtained from multiple herding households employing distinct caprine husbandry strategies for different production goals. Alternatively, the intensification of agricultural activities (suggested by the increased importance of cattle) necessitated an extended season of birth in order to reduce convergence of labor-intensive plowing of fields and planting new crops with equally labor-intensive lambing.

### Oxygen (δ^18^O) and carbon (δ^13^C) isotope results

**Fig 7:**
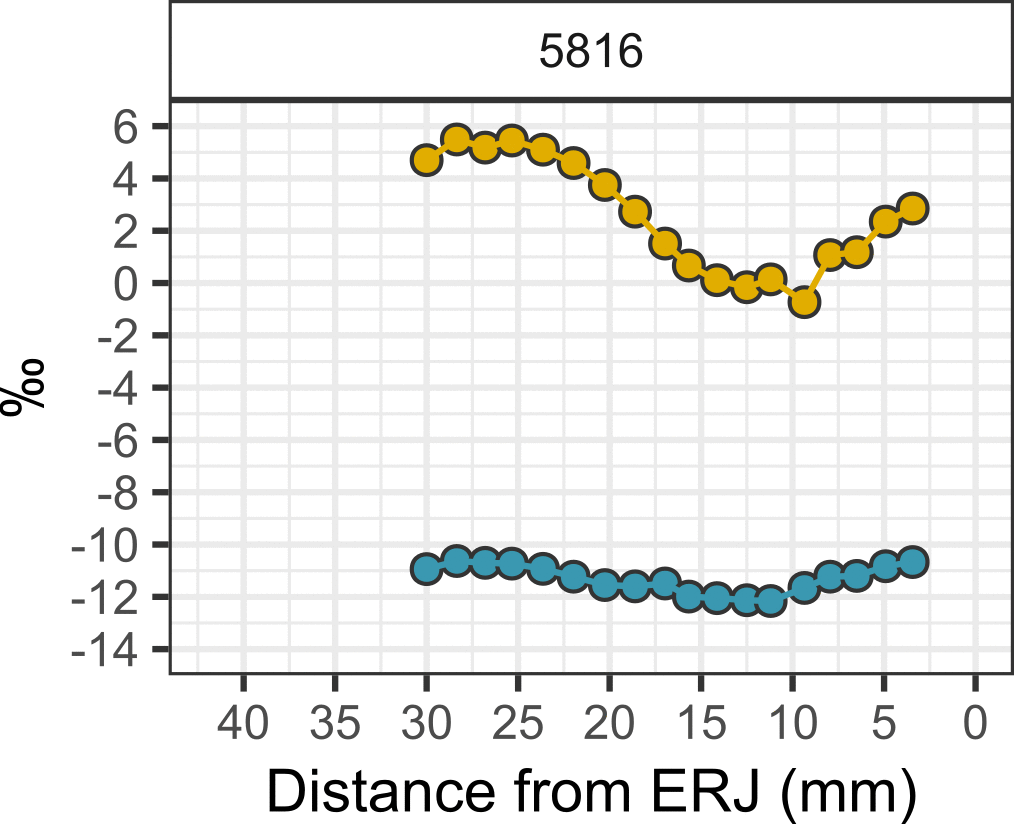
Intra-tooth oxygen (δ^18^O) and carbon (δ^13^C) isotope sequences measured from a single sheep (*Ovis aries*) tooth recovered from the Early IRIIA period deposits. Carbon isotope (δ^13^C) values = blue, oxygen isotope (δ^18^O) values = yellow.

**Fig 8:**
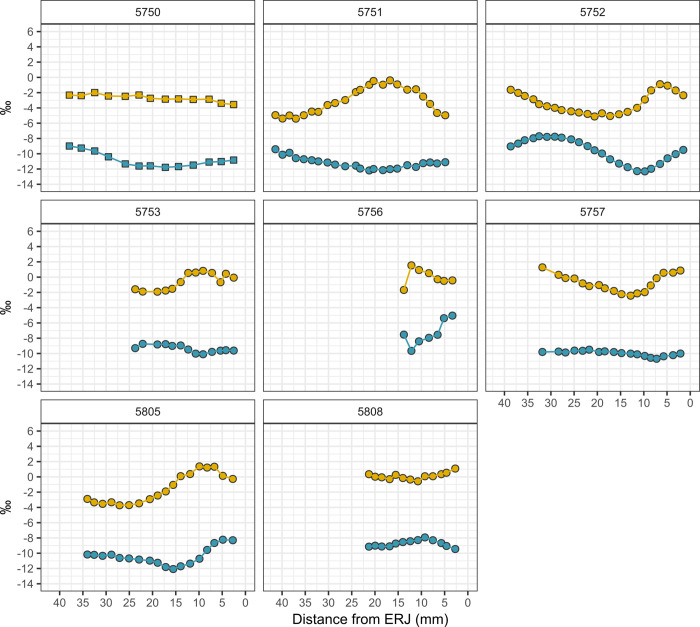
Intra-tooth oxygen (δ^18^O) and carbon (δ^13^C) isotope sequences measured for bovid taxa recovered from Late IRIIA period deposits. Squares = *Bos* sp., circles = Capra/Ovis (see Table 1 for detailed taxonomic assignments for caprines). Carbon isotope (δ^13^C) values = blue, oxygen isotope (δ^18^O) values = yellow.

**Fig 9:**
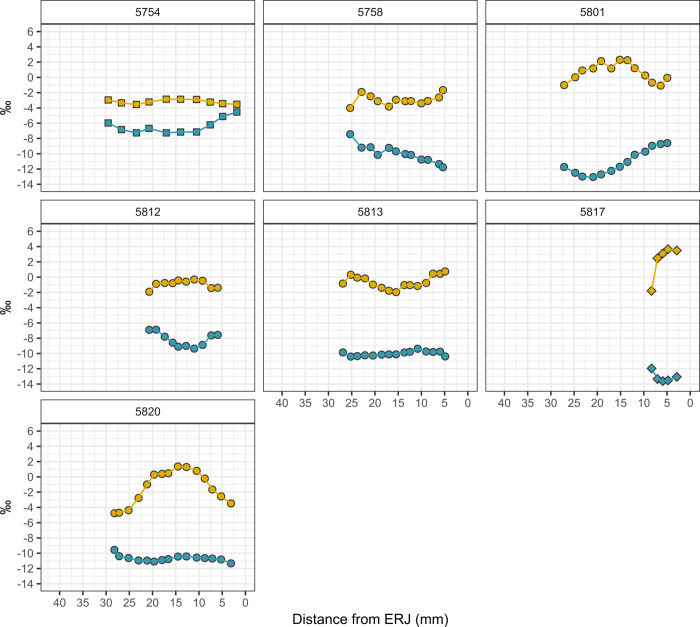
Intra-tooth oxygen (δ^18^O) and carbon (δ^13^C) isotope sequence measured from a single sheep (*Ovis aries*) tooth recovered from early IRIIB period deposits. Squares = *Bos* sp., circles = Capra/Ovis, diamonds = *Gazella* sp. (see Table 1 for detailed taxonomic assignments for caprines). Carbon isotope (δ^13^C) values = blue, oxygen isotope (δ^18^O) values = yellow.

The oxygen isotopic composition of the Hazor caprines reflects not only seasonal variation in geospatially sensitive meteoric water δ^18^O values, but also animal drinking and feeding behaviors that partially overwrite meteoric water oxygen isotope inputs [108]. Bovid livestock from Hazor exhibit moderate seasonal variation in oxygen isotopes expressed in sequentially sampled molars. Mean δ^18^O values yielded by each tooth specimen range from −3.4 to 2.6‰. Caprines from the IRIIA level range from −5.4 to 1.5‰ in δ^18^O while caprines from the IRIIB levels range from −4.8 to 3.7‰ (Table 4; S3 Table). Caprines exhibited moderate intra-tooth oxygen isotopic change (Δ^18^O) ranging from 2.7 to 5.0‰ in the IRIIA (Figs 7 and 8) and 2.4 to 6.1‰ in the IRIIB (Fig 9) and, in general, exhibited sinusoidal variation reflecting seasonal shifts in the oxygen isotope ratios of imbibed water. Cattle display low-amplitude intra-tooth oxygen isotope change, likely reflecting the intake by obligate drinking cattle of groundwater water from wells or springs, sources that yield an integrated isotopic signal derived from paleowaters, seasonal recharge events, recharge from different regional and local aquifers, or open water consistently affected by evaporative processes. The single IRIIA *Bos* specimen exhibits δ^18^O values ranging from −3.6 to −2.0‰, while the IRIIB *Bos* specimen exhibited a somewhat lower amplitude of oxygen isotopic change ranging from −3.6 to −2.9‰. The single gazelle specimen exhibited the highest intra-tooth δ^18^O values ranging from −1.8 to 3.7‰.

**Table 4:** Summary statistics for carbon (δ^13^C), oxygen (δ^18^O), and strontium (^87^Sr/^86^Sr) isotope values measured from sequentially sampled third mandibular molars from cattle (*Bos* cf. *taurus),* goat (C, *Capra hircus*), sheep (O, *Ovis aries*), sheep/goat (OC), and gazelle (*Gazella* sp.). ‘n’ indicates number of increments measured for each tooth, ∆ = indicates range of intra-tooth isotopic change (i.e., difference between minimum and maximum isotope values), asterisk (*) indicates specimens exhibiting a full seasonal cycle in oxygen isotope sequences. Strontium isotope values coincide with maximum and minimum δ^18^O values expressed within each tooth.

ASIL #	Taxon	Period	n	δ^13^C					δ^18^O						
				mean	stdev	min	max	Δ^13^C	mean	stdev	min	max	Δ^18^O	^87^Sr/^86^Sr (δ^18^O_max_)	^87^Sr/^86^Sr (δ^18^O_min_)
5816*	O	early IRIIA	18	−11.3	0.5	−12.2	−10.6	1.5*	2.6	2.2	−0.7	5.5	6.2*		
5750	Bos	late IRIIA	13	−10.8	1.0	−11.8	−9.0	2.8	−2.7	0.4	−3.6	−2.0	1.6	0.70659	0.70666
5751*	OC	late IRIIA	23	−11.2	0.7	−12.2	−9.4	2.8*	−3.1	1.8	−5.4	−0.4	5.0*	–	–
5752*	OC	late IRIIA	23	−9.7	1.6	−12.3	−7.7	4.6*	−3.4	1.4	−5.1	−0.9	4.2*	0.70598	0.70601
5753*	C	late IRIIA	13	−9.4	0.5	−10.1	−8.7	1.4*	−0.5	1.1	−1.9	0.8	2.7*	–	–
5756	C	late IRIIA	7	−7.4	1.6	−9.7	−5.0	4.6	0.0	1.1	−1.7	1.5	3.2	0.70765	0.70757
5757*	C?	late IRIIA	18	−10.0	0.3	−10.7	−9.5	1.2*	−0.7	1.2	−2.4	1.3	3.7*	0.70785	0.70780
5805*	O	late IRIIA	18	−10.4	1.1	−12.1	−8.2	3.8*	−1.6	1.9	−3.7	1.4	5.1*	0.70698	0.70722
5808	O	late IRIIA	13	−8.8	0.4	−9.5	−7.9	1.5	0.1	0.4	−0.6	1.1	1.7	0.70768	0.70747
5754	Bos	IRIIB	10	−6.4	1.0	−7.3	−4.5	2.7	−3.2	0.3	−3.6	−2.9	0.7	0.70850	0.70844
5758	OC	IRIIB	12	−10.0	1.2	−11.8	−7.4	4.3*	−2.9	0.7	−4.0	−1.7	2.4*	0.70776	0.70760
5801*	OC	IRIIB	13	−11.1	1.7	−13.1	−8.6	4.5*	0.7	1.2	−1.1	2.3	3.4*	0.70680	0.70749
5812	OC	IRIIB	10	−8.2	0.9	−9.4	−6.9	2.5	−0.9	0.5	−1.9	−0.3	1.6	–	–
5813*	OC	IRIIB	15	−10.0	0.3	−10.4	−9.4	1.0*	−0.6	0.8	−2.0	0.7	2.7*	0.70750	0.70791
5817	Gazella	IRIIB	5	−13.1	0.7	−13.6	−12.0	1.7	2.2	2.3	−1.8	3.7	5.5	0.70737	–
5820*	OC	IRIIB	15	−10.7	0.4	−11.3	−9.6	1.8*	−1.4	2.2	−4.8	1.4	6.1*	0.70680	0.70691

**Fig 10:**
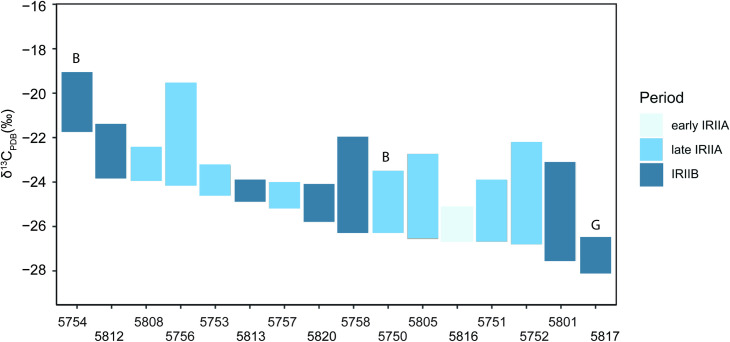
Range of intra-tooth δ^13^C values for Hazor caprines (boxes without labels), cattle (B), and gazelle (G). The periods are marked by color: light blue = Early IRIIA, blue = Late IRIIA, dark blue = IRIIB.

Caprines from Hazor yield a wide range of intra-tooth δ^13^C values from −12.2‰ to −10.6‰ observed in the single early IRIIA specimen, −12.3‰ to −5.0‰ for late IRIIA animals, and −13.6‰ to −6.9‰ for IRIIB animals (Fig 10). Caprines from both periods exhibited similarly wide variation in intra-tooth carbon isotopic change (Δ^13^C) ranging from +1.2‰ to 4.6‰ in the IRIIA (Table 4, Figs 7 and 8) and +1.0‰ to 4.5‰ in the IRIIB (Fig 9); carbon isotope sequences exhibit sinusoidal variation reflecting seasonal changes in dietary intake. The single *Bos* specimen from the late IRIIA level at Hazor exhibits moderate intra-tooth carbon isotopic variation ranging from −11.8 to −9.0‰ while the *Bos* specimen from the IRIIB level shows higher δ^13^C values from −7.3 to −4.5‰. Furthermore, intra-tooth carbon and oxygen isotopic variation exhibits consistent seasonal patterning between individuals. In general, seasonal shifts in intra-tooth carbon isotope change are inversely related to changes in oxygen isotopic composition, such that low wet-season δ^18^O values coincide with high δ^13^C values, while high summer season δ^18^O values correspond with low δ^13^C values (Figs 7−9). Other sequences instead exhibit high δ^13^C values associated with mid-range δ^18^O values likely representative of a spring season enamel formation (e.g., 5752), whereas another set of sequences show  low δ^13^C values corresponding to mid-range δ^18^O values expressed after summer maxima oxygen isotope values consistent with an autumn season formation period (e.g., 5805).

### Strontium isotope results

#### The range of variation of strontium isotope values.

**Fig 11:**
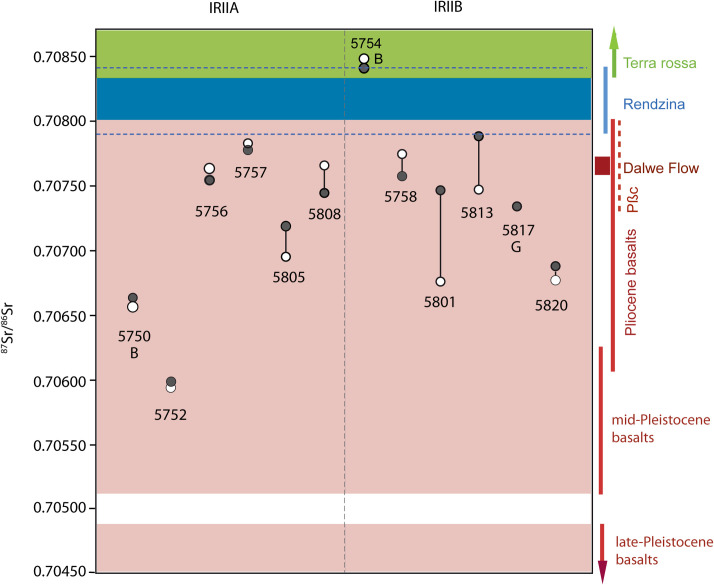
Strontium isotope values for Hazor caprines, cattle, and gazelle. Open circles coincide with δ^18^O_max_ values and black circles coincide with δ^18^O_min_ values. ‘B’ indicates cattle (*Bos* cf. *taurus*) and ‘G’ indicates gazelle (*Gazella* cf. *gazella*); all other values represent caprines. Colored horizontal bands and vertical bars indicate range of bioavailable strontium isotope values based on mean ± 1σ according to ref [63]. Horizontal blue dashed lines indicate the full range of ^87^Sr/^86^Sr values expected for rendzinas from refs [63,109].

The strontium isotopic composition of caprine livestock from both the IRIIA and IRIIB indicates these animals were pastured on basalt geologies (Table 4, Fig 11). A high proportion of strontium isotope ratios measured for animals from both periods ranged from approximately 0.7075 to 0.7079, values associated with Pliocene Dalton basalts, Dalwe basalts and cover basalts that cover the western and southeastern Golan, politically associated with Aram during the Iron Age II (Figs 2 and 3). Two IRIIA animals, one sheep (5752) and one cattle (5750) exhibited the very low strontium isotope ratios typically associated Pliocene basalts distributed to the east of the northern Hula Valley (Fig 3). Only one IRIIB cattle individual (5754; yielding radiogenic Sr values of 0.70844 and 0.70850) was grazed on terra rossa soils associated with Cretaceous limestones that covered the Lower Galilee region west and northwest of Hazor including the western edge of the Hula Valley, and also Jurassic limestones to the north-northeast. IRIIA and IRIIB bovids exhibited both low intra-tooth shifts in strontium isotope ratios suggestive of small-scale seasonal moves as well as wide intra-tooth differences in ^87^Sr/^86^Sr indicating larger scale movement to different pastures (Fig 11).

## Discussion

### Caprine pasturing systems unaffected by Iron Age conflicts

Multi-isotope data together suggest that bovid livestock grazed on spatially distinct pastures local to Hazor, within the Upper Jordan Valley, and also on more distant pastures located in the adjacent Golan Heights, an area that included contested landscapes held by Aram. Notably, there is no evidence that livestock pastured on rendzina soils located immediately north of Hazor or to the west in the Lower Galilee nor grazed on pastures supported by the radiogenic terra rossa soils that characterize the much of the Lower Galilee, the western portion of the UJV, or Mt. Hermon except for a single IRIIB cattle specimen (Fig 11). This individual (5754) exhibited the most radiogenic ^87^Sr/^86^Sr ratio (mean = 0.70846) of all sampled individuals from Hazor as well the highest dietary δ^13^C values (range = −21.9 to −19.0‰), indicating the animal regularly ingested water-stressed C_3_ plants and probably some C_4_ graze. This strontium value is expected for animals ingesting forage growing on terra rossa soils present along the western flanks of the Jordan Valley [63], which also supports ^13^C-enriched C_4_ sedges growing near springs and flowing water [32].

Instead, Hazor pasturing systems were oriented eastward during both the IRIIA and IRIIB, focused on the volcanic landscapes of the Golan Heights where well-watered pastures would have supplied herds with an abundant food supply. Pasture systems also included grazing grounds located to the south of Hazor in the rolling hills that surround the northern end of Lake Kinneret. Bioavailable ^87^Sr/^86^Sr ratios across these volcanic landscapes vary according to age with isotopic separation visible between plants growing on Pliocene, early Pleistocene, and late Pleistocene volcanic layers comprised of basalts and pyroclasts; this difference is driven largely by an increased contribution of atmospheric (strontium) deposition in soils that formed on old bedrock relative to younger bedrock [32].

The southern reaches of the UJV and much of the Golan Heights are comprised of Pliocene cover basalts (Pβc, 0.7074–0.7079) and Late Pliocene basalt flows (Dalwe basalts, 0.7077) (Fig 3; Dalwe values from ref. [109]). Both IRIIA (5756, 5757, and 5808) and IRIIB (5758, 5801, 5813, and 5817) caprines yielded strontium isotopes values, indicating animals were pastured in the Golan or to the south of Hazor where cover basalts are also present. Taking advantage of the pronounced elevation and precipitation gradients across the UJV and Golan, pasturing in either of these regions can be more finely resolved based on the oxygen isotope ratios recorded in discrete portions of the tooth enamel structure. The oxygen isotopic composition of meteoric precipitation and groundwater is influenced by altitude [110]. In the UJV and Golan region, there is a −0.26‰ decrease in meteoric water δ^18^O values per 100 m increase in elevation [111], similar to the global gradient of −0.28‰/100 m [38]. The precipitation ‘amount effect’ further modifies meteoric water oxygen isotope values with a decrease in precipitation levels corresponding with increasing meteoric water δ^18^O values [37]. Tel Hazor annually receives ca 450 mm of precipitation while the Golan Heights receives from 500 to 800 + mm per year.

We expect winter wet season δ^18^O_min_ values visible in intra-tooth sequences measured from archaeological caprines to more closely reflect the oxygen isotopic composition of open or groundwater sources which more closely reflect meteoric water oxygen isotope ratios, particularly under cooler, more humid conditions such as the wet season when evaporative enrichment is slowed. Furthermore, the isotopic composition of groundwater in the Cenomanian-Turanian limestone aquifer in northern Israel reflects meteoric water values in recharge areas [110]. Either one of these water sources would direct the oxygen isotopic composition of caprine body water during the wet season, in contrast to the later spring and summer seasons when ^18^O-enriched leaf water would also heavily influence bioapatite δ^18^O values of these semi-obligate drinkers [25,49]. Leaf water δ^18^O values are strongly influenced by evapotranspiration and air relative humidity [112]; these isotope values shift seasonally with leaf water present in foliar growth pushed out under cooler, more humid conditions during the wet season conditions more similar to meteoric water oxygen isotopes (i.e., depleted in ^18^O) compared to leaf water in later foliar growth enriched in ^18^O under more arid and warmer evaporative conditions [31,112].

Obligate-drinking cattle from Hazor, exhibiting an intra-tooth range from −3.6 to −2.0‰ in δ^18^O values, provide a rough estimate of seasonality in the oxygen isotope composition of groundwater and/or open water sources in the Upper Jordan Valley. However, the use of cattle δ^18^O values for the purposes of establishing an isotope reference data for local meteoric waters in the UJV is further complicated by the integrated contribution of precipitation with varied isotope compositions, imparted by amount and altitude effects that influence meteoric water δ^18^O values [104]. Variation in the oxygen isotopic composition of groundwater and open water sources within the Kinneret watershed adds further complexity [110]. In the case of Hazor, cattle δ^18^O values possibly reflect such an integrated signal at a sub-regional scale (i.e., Hula Valley) and may be more useful for establishing larger-scale inter-regional mobility or animal exchange within the diverse precipitation regimes of the southern Levant.

**Fig 12:**
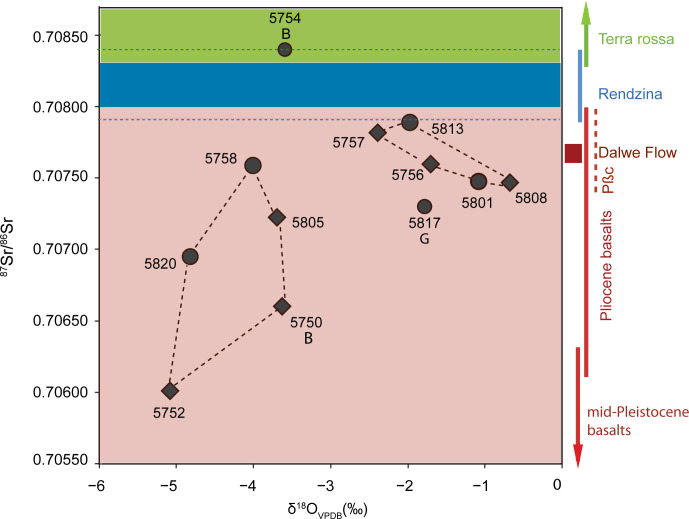
Intra-tooth δ^18^O_min_ values and paired strontium isotope values for Hazor caprines, cattle (B), and gazelle (G). Diamonds indicate IRIIA isotope values, circles indicate IRIIB isotope values. Convex hulls (dashed black lines) delineate livestock groups sharing a similar oxygen isotopic composition.

Livestock grazing on more radiogenic basalts associated with Pliocene cover basalts and Dalwe flow are significantly enriched +2.3‰ in δ^18^O (wet season minima values) relative to individuals foraging on less radiogenic Pliocene basalts (with the exception of individual 5758 feeding on more radiogenic basalts but yielding a low δ^18^O_min_ value; Fig 12; t-test p = 0.0185). This suggests that pastures supported by soils derived from cover basalts and Dalwe flow received ^18^O-enriched precipitation and, thus, likely located at lower elevations near Hazor. Individuals foraging on less radiogenic Pliocene basalts all exhibited low δ^18^O_min_ values consistent with grazing at higher elevations in the Golan. Individual 5758 likely also grazed at higher elevations in the Golan, albeit on pastures that supported more radiogenic soils.

δ^18^O_max_ values measured from tooth enamel appear to be less useful for establishing summer season mobility movement in semi-obligate drinking caprines in the absence of a large cohort of oxygen isotope data from contemporaneous non-obligate drinkers, such as gazelle, which would establish the upper limit of environmentally derived δ^18^O values from leaf water for a given locale [113]. Moderate oxygen isotopic enrichment of caprines relative to cattle, in particular summer season δ^18^O_max_ values (Table 4), confirms the steady contribution of an ^18^O-enriched water source to caprine body water, likely leaf water. The ingestion by caprines of evaporatively ^18^O-enriched leaf water, particularly during the warmer months, disrupts the geospatial relationship between meteoric water (and also groundwater) and oxygen isotope ratios recorded in tooth bioapatite. Consequently, the oxygen isotope ratios of obligate-drinking cattle and non-obligate drinking gazelle can be used to further constrain the geospatial origins of semi-obligate drinking caprines. For example, if the oxygen isotope ratios of caprines exhibiting strontium isotopes values associated with Upper Pleistocene basalts were higher than those yielded by the single gazelle from Hazor yielding an intra-tooth range of −1.8 to 3.7‰ in δ^18^O (and also yielding a strontium isotope value associated with Upper Pliocene basalts), it would be likely that the caprine came from a more arid region.

Altogether, intra-individual variation in δ^18^O_min_ values observed within each group of Hazor animals, distinguished by their strontium isotope ratios, is due to a complex intersection of environmental inputs, physiological effects, and dietary factors that modify the oxygen isotopic composition of body water. These include the isotopic composition of meteoric water and groundwater, seasonality in aridity levels, and foraging behaviour. Herbivores foraging on a higher proportion of graze exhibit higher δ^18^O values compared to browsers feeding in the same environment [25]. Shallow-rooting grasses exhibit pronounced evaporative ^18^O enrichment across the leaf surface [42], while deeper-rooted browse draws from ^18^O-depleted moisture in soils [114]. Consequently, differences in the relative contribution of C_3_ graze versus C_3_ browse to the caprine diet may drive variation in maxima δ^18^O values observed in Hazor caprines independent of animal mobility. Further complicating matters, animals foraging on well-watered green pastures, in particular new growth high in water content, obtain a larger proportion of their water from leaf water and compensate by reducing their intake from open water sources [115].

### Diversity in pasturing regimes confirmed by carbon isotopes

Hazor caprines grazed on open C_3_ Mediterranean pastures, indicated by moderately low dietary δ^13^C values exhibited by most IRIIA and IRIIB caprines, ranging from ca −26 to −21‰. The single gazelle (5817) foraged under a light Mediterranean forest canopy indicated by consistently low intra-tooth dietary δ^13^C values averaging −27.6‰ (Fig 10). Understory foliar growth under a forest canopy is depleted in ^13^C compared to flora growing in open landscapes due to recycling of ^13^C-depleted CO_2_ produced by decaying leaf litter [33,116], soil efflux [117], decreased photosynthesis rates [118], and low stomatal conductance under low light conditions [119].

Wide variation in caprine δ^13^C values, combined with differences in intra-tooth carbon isotopic patterning shared between IRIIA and IRIIB caprines, further confirms considerable spatial diversity in pasturing strategies (Fig 10). Low dietary δ^13^C_min_ values, ranging from −27.8 to −23.5‰ in both IRIIA and IRIIB caprines and coinciding with seasonal high δ^18^O_max_ values, indicate that these animals foraged during the spring and possibly early summer on well-watered C_3_ pastures. Seasonal transhumance from Hazor to higher elevation pastures located in the Golan Heights, a region that receives higher amounts of precipitation than the lower elevation Hula Valley due to orographic effects, would explain this isotopic pattern. Similarly low dietary δ^13^C values coinciding with high summer season δ^18^O values have been identified in Neolithic caprines east of the Jordan Valley; in this case, the pattern reflects seasonal vertical transhumance of animals to higher elevation, better watered C_3_ pastures in the Jordanian highlands [113]. At Hazor, winter season δ^13^C values were approximately 1–2.5‰ higher than summer season values, indicating ingestion of ^13^C-enriched fodder, a source that included later warm season C_3_ growth that grew under water-stressed conditions, perhaps collected from reserved pasture as agricultural by-products (i.e., straw or direct grazing on crop-stubble). Variation in the intensity and duration of fodder provided to Hazor sheep and goats, expressed as inter-individual differences in absolute δ^13^C values and shape of the carbon isotope sequences, most likely reflects differences in household access to fallow fields, winter pastures, and fodder storage capabilities.

Livestock were almost never grazed on drier, ^13^C-enriched pastures located in more water-deficient regions. Seasonal high dietary δ^13^C_max_ values of −19.0‰ and higher observed in one *Bos* (5754, IRIIB) and one caprine (5756, late IRIIA) indicate these animals ingested water-stressed graze or C_4_ sedges. Such pasturage was available in the Jordan Valley where ^13^C-enriched C_3_/C_4_ Irano-Turanian vegetation thrives. The pattern of high δ^13^C values coinciding with winter season minimum δ^18^O values suggests that these animals were moved during the winter months to extra-local pastures located in more arid environments and provided there with dried ^13^C-enriched graze in winter pastures or as fodder.

Overall, high δ^13^C values coinciding with δ^18^O_min_ values in Hazor caprines suggest that livestock were provisioned with a ^13^C-enriched fodder source, probably water-stressed graze collected during the summer months, during the coldest portion of the wet season (see further discussion below), while pronounced variation in caprine summer and winter season δ^13^C values, as well as inter-individual differences observed in their relationship with oxygen isotope minima and maxima values, indicates substantial diversity in pasturing and foddering strategies. These patterns suggest that the movement and provisioning of caprine herds were managed at the household level, rather than coordinated by a centralized authority, which would be expected to produce more homogenous isotopic patterning.

## Conclusion

Although isotopic analyses of a larger number of tooth specimens would help further define the ubiquity of specific caprine husbandry strategies practiced at Iron Age Hazor, the isotopic data presented here suggest that household-based flocks were grazed in spatially distinct pastures, independent of centralized coordination from Hazor. At the same time, the overall similarity in the heterogeneous character of pasturing regimes used by herders during periods of both calm and conflict indicates that local agro-pastoralist communities continued to pursue their own modes of subsistence and mobility despite pervasive inter-polity conflict between the Aramean and Israelite kingdoms. For sheep herders, the border between these polities remained permeable so that access to seasonal pastures was negotiated via channels that were independent of the state (Fig 1), for example according to tribal arrangements or other inter-community agreements. Conflicts may have instead been asserted between competing political entities in ways that primarily engaged urban elites and further acted out by military engagements. However, this did not involve close control of daily activities, including animal herding, conducted on hinterland landscapes. Ultimately, conflicts acted out by the military and ruling sectors of society did not affect the routine subsistence activities of everyday agro-pastoralists. The continued use of heterogeneous caprine pasturing strategies at Hazor through a period of settlement expansion, inter-polity conflict, and the transformation from an administrative center to a place more focused on domestic production speaks to the resilience of local agro-pastoralist populations.

## Supporting information

S1 Table Biometrical and fusion data for selected skeletal elements from Iron IIA and IIB Hazor. Metrical data according to von den Driesch (1976). (XLSX)

S2 Table Tooth wear data and epiphyseal fusion data aggregated by period for faunal specimens from Iron Age Hazor. (XLSX)

S3 Table Carbon (δ^13^C) and oxygen (δ^18^O) isotope values measured from the carbonate fraction of incrementally sampled bovid tooth specimens from Iron Age Hazor. All isotope values reported relative to VPDB. ERJ = enamel root junction.
(XLSX)

## References

[1] Parker BJ. Toward an understanding of borderland processes. Am antiq. 2006;71(1):77–100. doi: 10.2307/40035322

[2] Feuer B. Boundaries, Borders and Frontiers in Archaeology: A Study of Spatial Relationships. Jefferson, NC: McFarland & Company; 2016.

[3] Kolossov V, Scott J. Selected conceptual issues in border studies. belgeo. 2013;(1). doi: 10.4000/belgeo.10532

[4] Boozer AL. Frontiers and borderlands in imperial perspectives: exploring rome’s egyptian frontier. American Journal of Archaeology. 2013;117(2):275–92. doi: 10.3764/aja.117.2.0275

[5] González-Ruibal A. The cosmopolitan borderland: western Ethiopiac. AD 600–1800. Antiquity. 2021;95(380):530–48. doi: 10.15184/aqy.2021.23

[6] Glatz C, Casana J. Of highland-lowland borderlands: Local societies and foreign power in the Zagros-Mesopotamian interface. Journal of Anthropological Archaeology. 2016;44:127–47. doi: 10.1016/j.jaa.2016.09.001

[7] Knapp AB, Manning SW. Crisis in context: the end of the late bronze age in the eastern mediterranean. American Journal of Archaeology. 2016;120(1):99–149. doi: 10.3764/aja.120.1.0099

[8] Berlejung A. Outlook: Aramaeans outside of Syria: Palestine. In: Niehr H, editor. The Aramaeans in Ancient Syria. Leiden, Boston; 2014. p. 339–65.

[9] Mazar A. Archaeology of the Land of the Bible: 10,000 - 586 B.C.E. New York; 1992.

[10] Lemaire A. The boundary between the aramaean kingdom of damascus and the kingdom of Israel. In: Dušek J, Mynářová J, editors. Aramaean Borders: Defining Aramaean Territories in the 10th – 8th Centuries BCE. Leiden: Brill; 2019. p. 245–66.

[11] Finkelstein I. The Forgotten Kingdom: The Archaeology and History of Northern Israel. Atlanta: Society of Biblical Literature; 2013.

[12] Zwickel W. Borders between Aram-Damascus and Israel: A historical investigation. In: Dušek J, Mynářová J, editors. Aramaean Borders: Defining Aramaean Territories in the 10th – 8th Centuries BCE. Leiden, Boston. 2019; p. 267–335.

[13] Miller JM, Hayes JH. A history of ancient Israel and Judah. 2nd ed. Louisville, London; 2006.

[14] Ben-Tor A. Hazor: Canaanite metropolis, Israelite city. Jerusalem; 2015.

[15] Ben-Ami D. The early Iron Age II (Strata X–IX). In: Ben-Tor A, Ben-Ami D, Sandhaus D, editors. Hazor VI: The 1990-2009 Excavations, the Iron Age. Jerusalem. 2012. p. 52–153.

[16] Sergi O, Gadot Y. Omride palatial architecture as symbol in action: between state formation, obliteration, and heritage. Journal of Near Eastern Studies. 2017;76(1):103–11. doi: 10.1086/690651

[17] Sandhaus D. Hazor in the Ninth and Eight Centuries B.C.E. Near Eastern Archaeology. 2013;76:110–7.

[18] Wachtel I. The Upper Galilee during the Bronze and Iron Ages: Patterns of Settlement, Economy and Society. The Hebrew University of Jerusalem; 2018.

[19] Hartal M. Archaeological survey as a source for the history of the Golan. Qadmoniot. 2014;148:80–9.

[20] Stepansky Y. The periphery of Hazor during the Bronze Age, the Iron Age and the Persian period: A regional – archaeological study. Tel Aviv University; 1999.

[21] Hammer E. Local landscape organization of mobile pastoralists in southeastern Turkey. Journal of Anthropological Archaeology. 2014;35:269–88. doi: 10.1016/j.jaa.2014.06.001

[22] Makarewicz CA. Winter pasturing practices and variable fodder provisioning detected in nitrogen (δ15N) and carbon (δ13C) isotopes in sheep dentinal collagen. Journal of Archaeological Science. 2014;41:502–10. doi: 10.1016/j.jas.2013.09.016

[23] Arbuckle BS, Hammer EL. The rise of pastoralism in the ancient Near East. J Archaeol Res. 2018;27(3):391–449. doi: 10.1007/s10814-018-9124-8

[24] Makarewicz CA. Stable isotopes in pastoralist archaeology as indicators of diet, mobility, and animal husbandry practices. In: Ventresca Miller AR, Makarewicz CA, editors. Isotopic Investigations of Pastoralism in Prehistory. London: Routledge; 2018. p. 141–8.

[25] Makarewicz CA, Pederzani S. Oxygen (δ18O) and carbon (δ13C) isotopic distinction in sequentially sampled tooth enamel of co-localized wild and domesticated caprines: Complications to establishing seasonality and mobility in herbivores. Palaeogeography, Palaeoclimatology, Palaeoecology. 2017;485:1–15. doi: 10.1016/j.palaeo.2017.01.010

[26] Ventresca Miller AR, Bragina TM, Abil YA, Rulyova MM, Makarewicz CA. Pasture usage by ancient pastoralists in the northern Kazakh steppe informed by carbon and nitrogen isoscapes of contemporary floral biomes. Archaeol Anthropol Sci. 2018;11(5):2151–66. doi: 10.1007/s12520-018-0660-4

[27] Tieszen LL. Natural variations in the carbon isotope values of plants: Implications for archaeology, ecology, and paleoecology. Journal of Archaeological Science. 1991;18(3):227–48. doi: 10.1016/0305-4403(91)90063-u

[28] Smith BN, Epstein S. Two categories of c/c ratios for higher plants. Plant Physiol. 1971;47(3):380–4. doi: 10.1104/pp.47.3.380 16657626 PMC365873

[29] Araus JL, Villegas D, Aparicio N, del Moral LFG, El Hani S, Rharrabti Y, et al. Environmental factors determining carbon isotope discrimination and yield in durum wheat under mediterranean conditions. Crop Science. 2003;43(1):170–80. doi: 10.2135/cropsci2003.1700

[30] Ehleringer JR, Dawson TE. Water uptake by plants: perspectives from stable isotope composition. Plant Cell & Environment. 1992;15(9):1073–82. doi: 10.1111/j.1365-3040.1992.tb01657.x

[31] Flanagan LB, Ehleringer JR. Stable isotope composition of stem and leaf water: applications to the study of plant water use. Functional Ecology. 1991;5(2):270. doi: 10.2307/2389264

[32] Hartman G, Danin A. Isotopic values of plants in relation to water availability in the Eastern Mediterranean region. Oecologia. 2010;162(4):837–52. doi: 10.1007/s00442-009-1514-7 19956974 PMC2841277

[33] Vogel JC. Recycling CO2 in a forest environment. Oecologia Plantarum. 1978;13:89–94.

[34] Bonafini M, Pellegrini M, Ditchfield P, Pollard AM. Investigation of the ‘canopy effect’ in the isotope ecology of temperate woodlands. Journal of Archaeological Science. 2013;40(11):3926–35. doi: 10.1016/j.jas.2013.03.028

[35] Cerling TE, Harris JM. Carbon isotope fractionation between diet and bioapatite in ungulate mammals and implications for ecological and paleoecological studies. Oecologia. 1999;120(3):347–63. doi: 10.1007/s004420050868 28308012

[36] Gat JR. Oxygen and hydrogen isotopes in the hydrologic cycle. Annu Rev Earth Planet Sci. 1996;24(1):225–62. doi: 10.1146/annurev.earth.24.1.225

[37] Dansgaard W. Stable isotopes in precipitation. Tellus. 1964;16(4):436–68. doi: 10.1111/j.2153-3490.1964.tb00181.x

[38] Poage MA. Empirical relationships between elevation and the stable isotope composition of precipitation and surface waters: considerations for studies of paleoelevation change. American Journal of Science. 2001;301(1):1–15. doi: 10.2475/ajs.301.1.1

[39] Bajjali W. Spatial variability of environmental isotope and chemical content of precipitation in Jordan and evidence of slight change in climate. Appl Water Sci. 2012;2(4):271–83. doi: 10.1007/s13201-012-0046-1

[40] Sternberg LSL. Oxygen and hydrogen isotope ratios in plant cellulose: mechanisms and applications. In: Rundel PW, Ehleringer JR, Nagy KA, editors. Stable isotopes in ecological research. New York: Springer; 1989. p. 124–41.

[41] Yakir D. Variations in the natural abundance of oxygen-18 and deuterium in plant carbohydrates. Plant Cell & Environment. 1992;15(9):1005–20. doi: 10.1111/j.1365-3040.1992.tb01652.x

[42] Cernusak LA, Barbour MM, Arndt SK, Cheesman AW, English NB, Feild TS, et al. Stable isotopes in leaf water of terrestrial plants. Plant Cell Environ. 2016;39(5):1087–102. doi: 10.1111/pce.12703 26715126

[43] Cerling TE, Quade J. Stable carbon and oxygen isotopes in soil carbonates. In: Swart PK, Lohmann KC, McKenzie J, Savin S, editors. Climate Change in Continental Isotopic Records. Washington, D.C.: American Geophysical Union; 1993. p. 217–31.

[44] Dawson TE, Mambelli S, Plamboeck AH, Templer PH, Tu KP. Stable isotopes in plant ecology. Annu Rev Ecol Syst. 2002;33(1):507–59. doi: 10.1146/annurev.ecolsys.33.020602.095451

[45] Gonfiantini R, Gratziu S, Tongiogi E. Oxygen isotopic composition of water in leaves. Isotopes and Radiation in Soil-Plant Nutrition Studies. Proceedings of the symposium on the use of isotopes and radition agriculture organization. Vienna, Austria: International Atomic Energy Agency; 1965. p. 405–10.

[46] Farquhar GD, Cernusak LA. On the isotopic composition of leaf water in the non-steady state. Funct Plant Biol. 2005;32(4):293–303. doi: 10.1071/FP04232 32689132

[47] Kohn MJ. Carbon isotope compositions of terrestrial C3 plants as indicators of (paleo)ecology and (paleo)climate. Proc Natl Acad Sci U S A. 2010;107(46):19691–5. doi: 10.1073/pnas.1004933107 21041671 PMC2993332

[48] Sponheimer M, Lee-Thorp JA. Oxygen isotopes in enamel carbonate and their ecological significance. Journal of Archaeological Science. 1999;26(6):723–8. doi: 10.1006/jasc.1998.0388

[49] Levin NE, Cerling TE, Passey BH, Harris JM, Ehleringer JR. A stable isotope aridity index for terrestrial environments. Proc Natl Acad Sci U S A. 2006;103(30):11201–5. doi: 10.1073/pnas.0604719103 16840554 PMC1544065

[50] Helliker BR, Ehleringer JR. Grass blades as tree rings: environmentally induced changes in the oxygen isotope ratio of cellulose along the length of grass blades. New Phytol. 2002;155(3):417–24. doi: 10.1046/j.1469-8137.2002.00480.x 33873305

[51] Makarewicz CA, Sealy J. Dietary reconstruction, mobility, and the analysis of ancient skeletal tissues: Expanding the prospects of stable isotope research in archaeology. Journal of Archaeological Science. 2015;56:146–58. doi: 10.1016/j.jas.2015.02.035

[52] Balasse M, Ambrose SH, Smith AB, Price TD. The seasonal mobility model for prehistoric herders in the South-western cape of South Africa assessed by isotopic analysis of sheep tooth enamel. Journal of Archaeological Science. 2002;29(9):917–32. doi: 10.1006/jasc.2001.0787

[53] Evans J, Parker Pearson M, Madgwick R, Sloane H, Albarella U. Strontium and oxygen isotope evidence for the origin and movement of cattle at Late Neolithic Durrington Walls, UK. Archaeol Anthropol Sci. 2019;11(10):5181–97. doi: 10.1007/s12520-019-00849-w

[54] Alexander Bentley R. Strontium isotopes from the Earth to the archaeological skeleton: a review. J Archaeol Method Theory. 2006;13(3):135–87. doi: 10.1007/s10816-006-9009-x

[55] Ericson JE. Strontium isotope characterization in the study of prehistoric human ecology. Journal of Human Evolution. 1985;14(5):503–14. doi: 10.1016/s0047-2484(85)80029-4

[56] Capo RC, Stewart BW, Chadwick OA. Strontium isotopes as tracers of ecosystem processes: theory and methods. Geoderma. 1998;82(1–3):197–225. doi: 10.1016/s0016-7061(97)00102-x

[57] Slovak NM, Paytan A. Applications of Sr Isotopes in Archaeology.In Baskaran M, editor. Advances in Isotope Geochemistry. Springer Berlin Heidelberg; 2011. p. 743–68. doi: 10.1007/978-3-642-10637-8_35

[58] Retzmann A, Blanz M, Zitek A, Irrgeher J, Feldmann J, Teschler-Nicola M, et al. A combined chemical imaging approach using (MC) LA-ICP-MS and NIR-HSI to evaluate the diagenetic status of bone material for Sr isotope analysis. Anal Bioanal Chem. 2019;411(3):565–80. doi: 10.1007/s00216-018-1489-5 30511253

[59] Budd P, Montgomery J, Barreiro B, Thomas RG. Differential diagenesis of strontium in archaeological human dental tissues. Applied Geochemistry. 2000;15(5):687–94. doi: 10.1016/s0883-2927(99)00069-4

[60] Sneh A, Weinberger R. Geological Map of Israel. Jerusalem: Israel Geological Survey; 2014.

[61] Ganor E, Foner HA. Mineral dust concentrations, deposition fluxes and deposition velocities in dust episodes over Israel. J Geophys Res. 2001;106(D16):18431–7. doi: 10.1029/2000jd900535

[62] Herut B, Starinsky A, Katz A. Strontium in rainwater from Israel: Sources, isotopes and chemistry. Earth and Planetary Science Letters. 1993;120(1–2):77–84. doi: 10.1016/0012-821x(93)90024-4

[63] Hartman G, Richards M. Mapping and defining sources of variability in bioavailable strontium isotope ratios in the Eastern Mediterranean. Geochimica et Cosmochimica Acta. 2014;126:250–64. doi: 10.1016/j.gca.2013.11.015

[64] Spiro B, Ashkenazi S, Starinsky A, Katz A. Strontium isotopes in Melanopsis sp. as indicators of variation in hydrology and climate in the Upper Jordan Valley during the Early-Middle Pleistocene, and wider implications. J Hum Evol. 2011;60(4):407–16. doi: 10.1016/j.jhevol.2010.07.026 21036385

[65] Simpson B, Carmi I. The hydrology of the Jordan tributaries (Israel): Hydrographic and isotopic investigation. Journal of Hydrology. 1983;62(1–4):225–42. doi: 10.1016/0022-1694(83)90104-x

[66] Gur D, Bar-Matthews M, Sass E. Hydrochemistry of the main Jordan River sources: Dan, Banias, and Kezinim springs, north Hula Valley, Israel. Israel Journal of Earth Sciences. 2003;52(3–4):155–78. doi: 10.1560/rrmw-9wxd-31vu-mwhn

[67] Danin A. Distribution atlas of plants in the flora palaestina. Jerusalem: Israel Academy of Science and Humanities; 2004.

[68] Schiebel V, Litt T. Holocene vegetation history of the southern Levant based on a pollen record from Lake Kinneret (Sea of Galilee), Israel. Veget Hist Archaeobot. 2017;27(4):577–90. doi: 10.1007/s00334-017-0658-3

[69] Danin A. Flora and vegetation of Israel and adjacent areas. In: Yom-Tov Y, Tchernov E, editors. The zoogeography of Israel. Dordrecht; 1995. p. 129–59.

[70] Sapir-Hen L, Bar-Oz G, Sharon I, Gilboa A, Dayan T. Understanding faunal contexts of a complex Tell: Tel Dor, Israel, as a case study. Journal of Archaeological Science. 2012;39(3):590–601. doi: 10.1016/j.jas.2011.09.027

[71] Davis SJM. A rapid method for recording information about mammal bones from archaeological sites. Ancient Monuments Laboratory Reports. 1992;12:19–92.

[72] Marom N, Bar-Oz G. “Measure for measure”: a taphonomic reconsideration of the Kebaran site of Ein Gev I, Israel. Journal of Archaeological Science. 2008;35(2):214–27. doi: 10.1016/j.jas.2007.03.004

[73] Trentacoste A. Sometimes less is more: Comparison of rapid and traditional recording methods. University of Sheffield; 2009.

[74] Morin E, Ready E, Boileau A, Beauval C, Coumont M-P. Problems of identification and quantification in archaeozoological analysis, Part I: Insights from a blind test. J Archaeol Method Theory. 2016;24(3):886–937. doi: 10.1007/s10816-016-9300-4

[75] Silver IA. The ageing of domestic animals. In: Brothwell DR, Higgs ES, editors. Science in Archaeology. London: Thames & Hudson. 1963. p. 250–68.

[76] Payne S. Kill-off patterns in sheep and goats: the mandibles from Aşvan Kale. Anatol Stud. 1973;23:281–303. doi: 10.2307/3642547

[77] Davis SJM. Measurements of a group of adult female shetland sheep skeletons from a single flock: a baseline for zooarchaeologists. Journal of Archaeological Science. 1996;23(4):593–612. doi: 10.1006/jasc.1996.0056

[78] von den Driesch A. A guide to the measurement of animal bones from archaeological sites. Cambridge, MA: Peabody Museum; 1976.

[79] Meyer D, Zeileis A, Hornik K. vcd: Visualizing Categorical Data. R package version 1.4-7; 2020.

[80] Pieterse JK. Fitting curves to your data using least squares. JKP: Application Development Services. 2020. https://jkp-ads.com/Articles/leastsquares.asp

[81] Milhaud G, Nezit J. Développement des molaires chez le mouton. Étude morphologique, radiographique et microdurométrique. Recueil de Medecine Veterinaire. 1991;167:121–7.

[82] Weinreb MM, Sharav Y. Tooth development in sheep. Am J Vet Res. 1964;25:891–908. 14266896

[83] Kierdorf H, Witzel C, Upex B, Dobney K, Kierdorf U. Enamel hypoplasia in molars of sheep and goats, and its relationship to the pattern of tooth crown growth. J Anat. 2012;220(5):484–95. doi: 10.1111/j.1469-7580.2012.01482.x 22352403 PMC3403278

[84] Tornero C, Bălăşescu A, Ughetto-Monfrin J, Voinea V, Balasse M. Seasonality and season of birth in early Eneolithic sheep from Cheia (Romania): methodological advances and implications for animal economy. Journal of Archaeological Science. 2013;40(11):4039–55. doi: 10.1016/j.jas.2013.05.013

[85] Brown WA, Christofferson PV, Massler M, Weiss MB. Postnatal tooth development in cattle. Am J Vet Res. 1960;21:7–34. 13805043

[86] Haber A, Dayan T. Analyzing the process of domestication: Hagoshrim as a case study. Journal of Archaeological Science. 2004;31(11):1587–601. doi: 10.1016/j.jas.2004.04.001

[87] Halstead P, Collins P, Isaakidou V. Sorting the sheep from the goats: morphological distinctions between the mandibles and mandibular teeth of adult ovis and capra. Journal of Archaeological Science. 2002;29(5):545–53. doi: 10.1006/jasc.2001.0777

[88] Brookman TH, Ambrose SH. Seasonal variation in kangaroo tooth enamel oxygen and carbon isotopes in southern Australia. Quat res. 2012;78(2):256–65. doi: 10.1016/j.yqres.2012.05.011 22915330

[89] Berthon R, Kovačiková L, Tresset A, Balasse M. Integration of Linearbandkeramik cattle husbandry in the forested landscape of the mid-Holocene climate optimum: Seasonal-scale investigations in Bohemia. Journal of Anthropological Archaeology. 2018;51:16–27. doi: 10.1016/j.jaa.2018.05.002

[90] Reade H, Stevens RE, Barker G, O’Connell TC. Tooth enamel sampling strategies for stable isotope analysis: Potential problems in cross-method data comparisons. Chemical Geology. 2015;404:126–35. doi: 10.1016/j.chemgeo.2015.03.026

[91] Suga S. Progressive mineralization pattern of developing enamel during the maturation stage. J Dent Res. 1982;Spec No:1532–42. 6958712

[92] Smith CE. Cellular and chemical events during enamel maturation. Crit Rev Oral Biol Med. 1998;9(2):128–61. doi: 10.1177/10454411980090020101 9603233

[93] Simmer JP, Richardson AS, Hu Y-Y, Smith CE, Ching-Chun Hu J. A post-classical theory of enamel biomineralization… and why we need one. Int J Oral Sci. 2012;4(3):129–34. doi: 10.1038/ijos.2012.59 22996272 PMC3464985

[94] Green DR, Green GM, Colman AS, Bidlack FB, Tafforeau P, Smith TM. Synchrotron imaging and Markov Chain Monte Carlo reveal tooth mineralization patterns. PLoS One. 2017;12(10):e0186391. doi: 10.1371/journal.pone.0186391 29049333 PMC5648163

[95] Passey BH, Cerling TE. Tooth enamel mineralization in ungulates: implications for recovering a primary isotopic time-series. Geochimica et Cosmochimica Acta. 2002;66(18):3225–34. doi: 10.1016/s0016-7037(02)00933-x

[96] Balasse M, Boury L, Ughetto-Monfrin J, Tresset A. Stable isotope insights (δ18O,δ13C) into cattle and sheep husbandry at Bercy (Paris, France, 4th millennium BC): birth seasonality and winter leaf foddering. Environmental Archaeology. 2012;17(1):29–44. doi: 10.1179/1461410312z.0000000003

[97] Zazzo A, Bendrey R, Vella D, Moloney AP, Monahan FJ, Schmidt O. A refined sampling strategy for intra-tooth stable isotope analysis of mammalian enamel. Geochimica et Cosmochimica Acta. 2012;84:1–13. doi: 10.1016/j.gca.2012.01.012

[98] Montgomery J, Evans JA, Horstwood MSA. Evidence for long-term averaging of strontium in bovine enamel using TIMS and LA-MC-ICP-MS strontium isotope intra-molar profiles. Environmental Archaeology. 2010;15(1):32–42. doi: 10.1179/146141010x12640787648694

[99] Bendrey R, Vella D, Zazzo A, Balasse M, Lepetz S. Exponentially decreasing tooth growth rate in horse teeth: implications for isotopic analyses. Archaeometry. 2014;57(6):1104–24. doi: 10.1111/arcm.12151

[100] Balasse M. Potential biases in sampling design and interpretation of intra-tooth isotope analysis. Intl J of Osteoarchaeology. 2003;13(1–2):3–10. doi: 10.1002/oa.656

[101] Pellegrini M, Snoeck C. Comparing bioapatite carbonate pre-treatments for isotopic measurements: Part 2 — Impact on carbon and oxygen isotope compositions. Chemical Geology. 2016;420:88–96. doi: 10.1016/j.chemgeo.2015.10.038

[102] Blaise E, Balasse M. Seasonality and season of birth of modern and late Neolithic sheep from south-eastern France using tooth enamel δ18O analysis. Journal of Archaeological Science. 2011;38(11):3085–93. doi: 10.1016/j.jas.2011.07.007

[103] Balasse M, Obein G, Ughetto-monfrin J, Mainland I. Investigating seasonality and season of birth in past herds: a reference set of sheep enamel stable oxygen isotope ratios. Archaeometry. 2011;54(2):349–68. doi: 10.1111/j.1475-4754.2011.00624.x

[104] Tornero C, Balasse M, Molist M, Saña M. Seasonal reproductive patterns of early domestic sheep at Tell Halula (PPNB, Middle Euphrates Valley): Evidence from sequential oxygen isotope analyses of tooth enamel. Journal of Archaeological Science: Reports. 2016;6:810–8. doi: 10.1016/j.jasrep.2015.10.038

[105] Zeder MA, Lapham HA. Assessing the reliability of criteria used to identify postcranial bones in sheep, Ovis, and goats, Capra. Journal of Archaeological Science. 2010;37(11):2887–905. doi: 10.1016/j.jas.2010.06.032

[106] Zeder MA. Understanding urban process through the study of specialized subsistence economy in the Near East. Journal of Anthropological Archaeology. 1988;7(1):1–55. doi: 10.1016/0278-4165(88)90006-2

[107] Langgut D, Finkelstein I, Litt T, Harald Neumann F, Stein M. Vegetation and climate changes during the bronze and iron ages (∼3600–600 BCE) in the Southern levant based on palynological records. Radiocarbon. 2015;57(2):217–35. doi: 10.2458/azu_rc.57.18555

[108] Makarewicz CA, Kolska-Horwitz L, Schwarcz H. Geospatial distinction in seasonal carbon isotopes (δ13C) but not oxygen isotopes (δ18O) in caprine bioapatite.

[109] Arnold ER, Greer JS, Ilan D, Thareani Y, Hartman G. “Come, O pilgrim”—but buy local: an isotopic investigation of animal provisioning at Iron Age II Tel Dan. Archaeol Anthropol Sci. 2021;13(4). doi: 10.1007/s12520-021-01291-7

[110] Gat JR, Dansgaard W. Stable isotope survey of the fresh water occurrences in Israel and the Northern Jordan Rift Valley. Journal of Hydrology. 1972;16(3):177–211. doi: 10.1016/0022-1694(72)90052-2

[111] Brielmann H. Recharge and discharge mechanism and dynamics in the mountainous northern Upper Jordan River catchment, Israel. 2008.

[112] Cernusak LA, Barbeta A, Bush RT, Eichstaedt Bögelein R, Ferrio JP, Flanagan LB, et al. Do 2 H and 18 O in leaf water reflect environmental drivers differently? New Phytol. 2022;235(1):41–51. doi: 10.1111/nph.18113 35322882 PMC9322340

[113] Makarewicz CA. Sequential δ13C and δ18O analyses of early Holocene bovid tooth enamel: Resolving vertical transhumance in Neolithic domesticated sheep and goats. Palaeogeography, Palaeoclimatology, Palaeoecology. 2017;485:16–29. doi: 10.1016/j.palaeo.2017.01.028

[114] Hirl RT, Schnyder H, Ostler U, Schäufele R, Schleip I, Vetter SH, et al. The 18 O ecohydrology of a grassland ecosystem – predictions and observations. Hydrol Earth Syst Sci. 2019;23(6):2581–600. doi: 10.5194/hess-23-2581-2019

[115] Jaber L, Chedid M, Hamadeh S. Water stress in small ruminants. Responses of Organisms to Water Stress. InTech. 2013. doi: 10.5772/53584

[116] Buchmann N, Brooks JR, Ehleringer JR. Predicting daytime carbon isotope ratios of atmospheric CO2 within forest canopies. Functional Ecology. 2002;16(1):49–57. doi: 10.1046/j.0269-8463.2001.00591.x

[117] Ehleringer JR, Buchmann N, Flanagan LB. Carbon isotope ratios in belowground carbon cycle processes. Ecological Applications. 2000;10(2):412–22. doi: 10.1890/1051-0761(2000)010[0412:ciribc]2.0.co;2

[118] Rijkers T, Pons TL, Bongers F. The effect of tree height and light availability on photosynthetic leaf traits of four neotropical species differing in shade tolerance. Functional Ecology. 2000;14(1):77–86. doi: 10.1046/j.1365-2435.2000.00395.x

[119] van der Merwe NJ, Medina E. The canopy effect, carbon isotope ratios and foodwebs in amazonia. Journal of Archaeological Science. 1991;18(3):249–59. doi: 10.1016/0305-4403(91)90064-v

[120] Moffat I, Rudd R, Willmes M, Mortimer G, Kinsley L, McMorrow L, et al. Bioavailable soil and rock strontium isotope data from Israel. Earth Syst Sci Data. 2020;12(4):3641–52. doi: 10.5194/essd-12-3641-2020

